# Gymnastics Experience Enhances the Development of Bipedal-Stance Multi-Segmental Coordination and Control During Proprioceptive Reweighting

**DOI:** 10.3389/fpsyg.2021.661312

**Published:** 2021-04-15

**Authors:** Albert Busquets, Blai Ferrer-Uris, Rosa Angulo-Barroso, Peter Federolf

**Affiliations:** ^1^Institut Nacional d'Educació Física de Catalunya (INEFC), Universitat de Barcelona (UB), Barcelona, Spain; ^2^Department of Kinesiology, California State University, Northridge, CA, United States; ^3^Department of Sport Science, University of Innsbruck, Innsbruck, Austria

**Keywords:** movement coordination, motor control, posture, sensory integration, children, adults, gymnastics, principal component analysis

## Abstract

Performance and control of upright bipedal posture requires a constant and dynamic integration of relative contributions of different sensory inputs (i. e., sensory reweighting) to enable effective adaptations as individuals face environmental changes and perturbations. Children with gymnastic experience showed balance performance closer to that of adults during and after proprioceptive alteration than children without gymnastic experience when their center of pressure (COP) was analyzed. However, a particular COP sway can be achieved through performing and coordinating different postural movements. The aim of this study was to assess how children and adults of different gymnastic experience perform and control postural movements while they have to adjust balance during and after bilateral tendon vibration. All participants were equipped with spherical markers attached to their skin and two vibrators strapped over the Achilles tendons. Bipedal stance was performed in three 45-s trials in two visual conditions (eyes open, EO, and eyes closed, EC) ordered randomly in which vibration lasted 10 s. Posture movements were analyzed by a principal component analysis (PCA) calculated on normalized and weighted markers coordinates. The relative standard deviation of each principal movement component (principal position, PP-rSTD) quantified its contribution to the whole postural movements, i.e., quantified the coordinative structure. The first (principal velocities, PV-rSTD) and second (principal accelerations, PA-rSTD) time-derivatives characterized the rate-dependent sensory information associated with and the neuromuscular control of the postural movements, respectively. Children without gymnastic experience showed a different postural coordinative structure and different sensory-motor control characteristics. They used less ankle movements in the anterior-posterior direction but increased ankle movements in medio-lateral direction, presented larger hip and trunk velocities, and exhibited more hip actions. Gymnastic experience during childhood seemed to benefit the development of proprioceptive reweighting processes in children, leading to a more mature form of coordinating and controlling posture similarly to adults.

## Introduction

Upright bipedal stance is one of the most common postures used in daily life activities but also in recreational and sports skills. Far from its simple appearance, performance, and control of a vertical posture on both feet requires constant adjustments to environmental changes and organism perturbations (Walter, 1998; Hatzitaki et al., 2002). These adjustments involve dynamic processes that require the integration of sensory information from multiple sources (e.g., visual, proprioceptive, and vestibular systems) and the use of coordinated muscular actions to control the body movements that preserve the desired posture (i.e., postural movements) (Forssberg and Nashner, 1982; Jeka et al., 2000; Peterka and Loughlin, 2004; Hsu et al., 2007; Peterka, 2018). In addition, several studies (Peterka, 2002, 2018; Carver et al., 2006; Assländer and Peterka, 2014; Hwang et al., 2014) demonstrated that a critical sensory process to achieve posture stability is the individual's capacity to increase or decrease the relative contribution of different sensory source inputs when availability and/or reliability of other sources change (i.e., sensory reweighting ability). Given that a sudden manipulation of the sensory sources typically leads to a loss of accuracy in the estimation of the body position (Jeka et al., 2004), the reweighting processes have to be performed fast enough to sufficiently modify the postural movements and return as soon as possible to the desired state of balance (i.e., stabilize balance) (Peterka, 2018). A common experimental approach to study the sensory reweighting processes is to expose individuals to a perturbation altering the sensory inputs and then remove such perturbation while measuring the postural behavior, which is usually characterized by the center of pressure (COP) trajectory (Vuillerme et al., 2001b; Jeka et al., 2004; Gautier et al., 2008a,b; McKay et al., 2014). Previous studies analyzing COP during bipedal stance were able to show the impact of the availability of different sensory sources and also confirmed that stabilization after proprioceptive manipulation (i.e., vibration) occurs not immediately, but gradually (Vuillerme et al., 2001b; McKay et al., 2014; Busquets et al., 2018).

Postural balance could be affected by multiple intrinsic factors but one of the main factors that modifies postural control ability in healthy individuals seems to be related to their motor experiences (Paillard, 2017). Motor experiences can be characterized by age (Peterka and Black, 1990; Hirabayashi and Iwasaki, 1995; Barela et al., 2003; Peterson et al., 2006; Cuisinier et al., 2011), as an estimator of the amount of experience performing the bipedal stance in domestic and leisure physical activities. In addition, motor experiences might further be influenced by the individual's deliberate practice experience in a sport with high balance and postural control demands, for example, gymnastics (Marin et al., 1999; Vuillerme et al., 2001a,b; Gautier et al., 2008a,b; Lamoth et al., 2009).

The ability to control independent bipedal stance and perform counteracting actions in face of balance perturbations is achieved early in childhood (Hadders-Algra, 2005; Chen et al., 2008). However, development of various components of postural control continues until adolescence (Assaiante, 2012; Verbecque et al., 2016). Especially, components related to the integration and reweighting of sensory information appears to need more experience time to resemble an adult-like structure (Peterka and Black, 1990; Hirabayashi and Iwasaki, 1995; Cuisinier et al., 2011; Assaiante, 2012; McKay et al., 2014; Busquets et al., 2018). When proprioceptive alteration with motor vibration is used to evaluate the sensory reweighting ability, it has been proposed that a significant development of the sensory integration ability occurs in children around 10 years of age (Cuisinier et al., 2011; McKay et al., 2014). However, adult-like levels of use and integration of the sensory information is not achieved until the age of 12–15 years (Peterka and Black, 1990; Hirabayashi and Iwasaki, 1995; Barela et al., 2003; Peterson et al., 2006).

Accumulation of deliberate experience in sport could enhance postural control including its entailed reweighting processes (Marin et al., 1999; Lamoth et al., 2009; Hrysomallis, 2011; Kiers et al., 2013). The high balance requirements to perform complex acrobatics and balance recovery after their completion exhibited in gymnastics motivated researchers to study its effects on bipedal stance performance and control (Vuillerme et al., 2001a,b; Gautier et al., 2008a,b; Busquets et al., 2018). Studies in adults showed that gymnastic experience did not enhance the reweighting processes or change the relative contribution of the sensory sources during the sensory manipulation of vision (i.e., optical flow) (Gautier et al., 2008b) or of proprioception (i.e., vibration applied on the Achilles tendon) (Vuillerme et al., 2001b; Busquets et al., 2018). Controversial results were obtained when re-integration of the manipulated sensory source was necessitated after the perturbation. Gautier et al. (2008a) and Vuillerme et al. (2001b) found that the gymnast groups stabilized COP sway faster than the non-gymnasts after optical flow and vibration manipulation had finished, respectively; in contrast, Busquets et al. (2018) reported no benefits from gymnastic experience in COP variables after vibration for adults. In children, Busquets et al. (2018) found evidence that gymnastic practice benefited postural control during and after proprioceptive perturbation (i.e., vibration applied on the Achilles tendon), bringing these children's performance closer to adult-like postural behavior. Their findings suggested that children's sensory reweighting capacity after proprioceptive information manipulation was not fully developed but gymnastic practice reduced the gap difference between children and adults.

Previous studies focusing on the age and/or gymnastic experience effects on the reweighting processes involved in bipedal standing utilized the COP sway characteristics as the primary outcome. The variables derived from COP summarize whole body movement changes in 2-dimensions (i.e., anterior –posterior, AP, and mediolateral, ML, directions). However, the human body is a multi-segmental system that could perform and coordinate postural movements in different ways in order to control the body sway (Kuo and Zajac, 1993; Hsu et al., 2007; Federolf, 2016). It was suggested that movements from ankle and hip joints could fully explain the postural adjustments required to control the body sway in bipedal standing (Nashner and McCollum, 1985; Runge et al., 1999; Afschrift et al., 2016), but recent studies presented evidence that other multi-segmental movements (i.e., postural movements) should be considered (Alexandrov et al., 2005; Pinter et al., 2008; Federolf et al., 2013). A data-driven principal component analysis (PCA), in which no variables are preselected, has been applied in previous research to study individuals' multi-segment postural movement components (Daffertshofer et al., 2004; Federolf et al., 2012, 2013). PCA provides multiple 1-dimensional whole body movement components (i.e., principal movements, PM) that together form the postural coordinative structure of the original movement. Changes in the relative contribution of the PM over time and their mathematical derivatives (i.e., velocity and acceleration) were proposed to describe how individuals perform and control their posture (Federolf, 2016; Haid et al., 2018; Longo et al., 2019).

Therefore, the analysis of the posture movements through a PCA would be an interesting approach to (1) better understand how the system achieves and maintains postural stability through integrating and reweighting inputs of the sensory sources and (2) to assess possible beneficial effects of age and gymnastic experience on individuals' balance ability. The purpose of this study was to investigate how children and adults perform and control postural movements to maintain bipedal standing when they deal with proprioceptive manipulations (bilateral ankle vibration) in two visual conditions (i.e., eyes open, EO, and eyes closed, EC). In addition to this age effect, the possible effect of gymnastic experience on these sensory reweighting processes to adjust standing posture was examined. We hypothesized that:

- Different movement amplitude (i.e., performance) and control of posture will emerge in individual movement components or in all of them when sensory information is altered. In addition, re-integration of the proprioceptive information will show gradual (not immediate) recovery to the pre-vibration coordinative structure, the sensory integration state, and to pre-vibration motor control characteristics. Larger differences will be found in the most demanding condition (i.e., EC + vibration).
- Children will show larger differences than adults in the coordinative structure, the sensory integration state, and the motor control variables in comparison with their before vibration values, especially when they re-integrate proprioceptive information. In addition, these possible changes will last longer in children than in adults.
- Gymnastic experience will provide individuals with a refined coordinative structure, sensory integration state, and motor control that allow them to better stabilize posture during the reweighting processes. Larger differences will be observed between children's groups while more subtle differences will be found between the adults' groups. Also, gymnastic experience will help children to develop an adult-like postural performance and control during the reweighting processes.

## Methods

### Participants

Seventy-seven participants from two cohorts (children and adults) were involved in this study (Table 1). Individuals 8–11 years of age were included in the child cohort (C) because at this age the proprioceptive ability is relatively stable to developmental changes (Goble et al., 2005; Deconinck et al., 2007). The adult cohort (A) was composed of participants of 15 years of age or older as previous research suggested that an adult-like balance is shown after 12 years of age (Peterson et al., 2006). Both cohorts were divided into two groups according to their gymnastic experience. Participants that practiced gymnastics to compete from regional to international championship levels, were part of the gymnast groups (G); while non-gymnast groups (NG) were formed by active individuals engaged in non-competitive organized sport activities. Individually reported training hours per week and competition history were used to classify participants. All participants declared to be healthy without disorders that could affect postural control at the time of the study. All participants or their legal guardians were fully informed and they signed a written consent form to participate in the study. In addition, young participants (<18 yrs) signed an assent form. The study was approved by the local ethics committee.

**Table 1:** Participants general characteristics.

	**Children**		**Adults**	
	**NG**	**G**	**NG**	**G**
	**M ± SD**	**M ± SD**	**M ± SD**	**M ± SD**
Female (*n*)/Male (n)	6/5	16/15	6/7	10/12
Age (yrs)	9.6 ± 1.1	9.2 ± 1.2	17.0 ± 1.3	16.3 ± 2.6
Experience (yrs)	–	3.2 ± 1.6	–	8.4 ± 3.4
Training (h/week)	1.75 ± 1.41	10.87 ± 4.65	5.35 ± 1.69	22.57 ± 8.12
Height (m)	1.41 ± 0.06	1.30 ± 0.08	1.70 ± 0.10	1.63 ± 0.09
Weight (kg)	35.44 ± 5.61	28.43 ± 5.66	66.31 ± 17.64	56.11 ± 9.94

*NG, non-gymnasts; G, gymnasts; M, mean; SD, standard deviation*.

### Experimental Protocol

**Figure 1:**
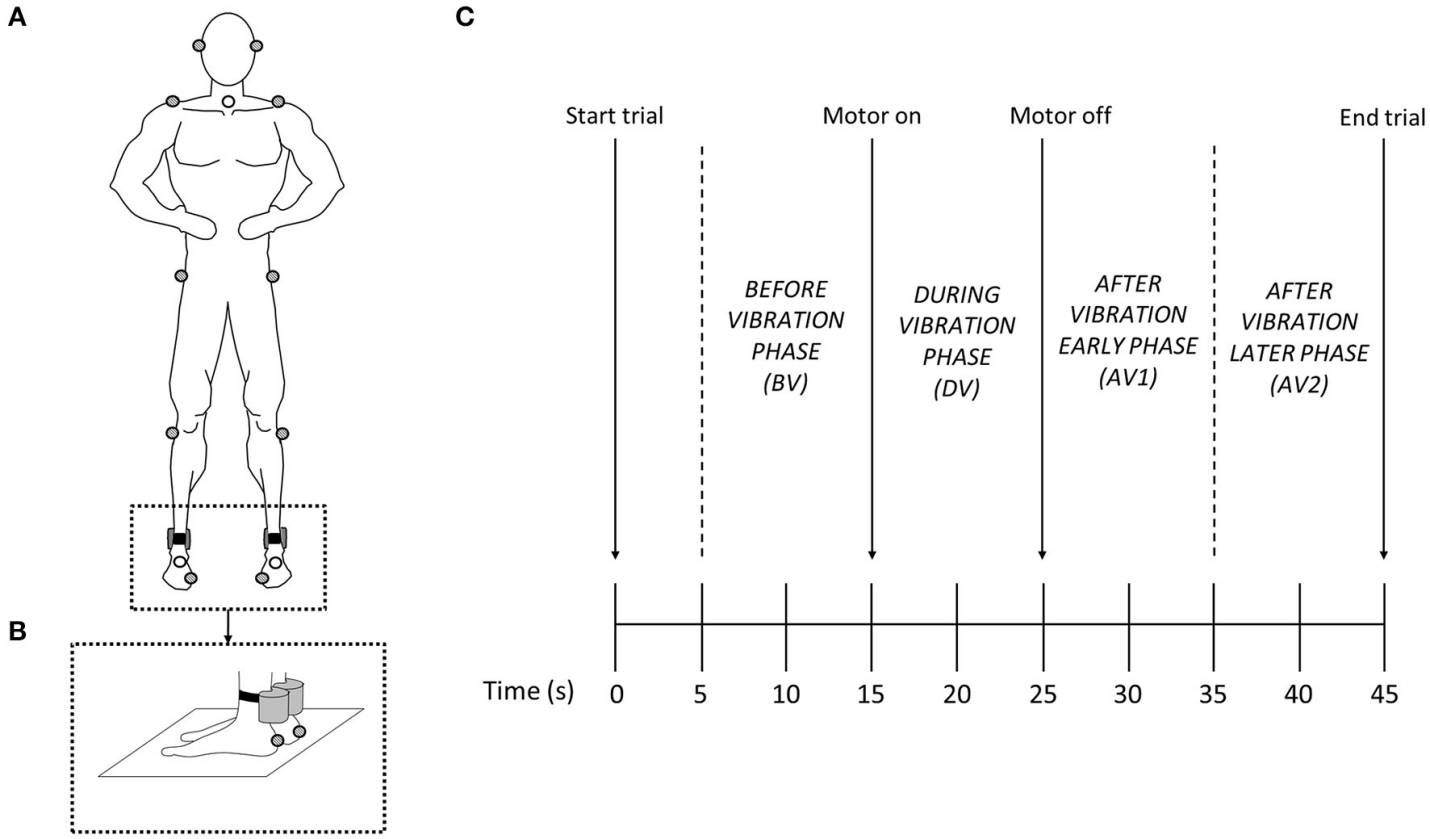
**(A)** Sketch of the body landmark marker dispositions. **(B)** Detail of the vibrator collocations. **(C)** 45-s trial timeline indicating when vibration was applied to the participant (motor on—motor off) and the 10-s phases used to calculate variables.

Participants stood barefoot and with both hands on their hips. The same base of support area across trials was maintained by asking participants to place their feet on their individual foot marks drawn on a piece of paper set on the floor during trial 1. To perturb the ankle proprioceptive system, the Achilles tendons of the right and left leg were stimulated with two vibrators (frequency = 85 Hz, amplitude = 1 mm) strapped over the tendons (Tjernström et al., 2006) (Figure 1). Recordings of simultaneous activation and deactivation of the vibrators during trials were computer synchronized with the motion capture system. In order to reduce startling responses, participants were allowed to experience vibration during 4–5 s before the start of the experimental protocol to familiarize themselves with the vibrators' effects.

Participants were asked to stand as steady as possible no matter what happened during the trials. Two visual conditions of three 45 s trials were performed during the session: eyes open (EO) and eyes closed (EC). In the EO condition the participants looked at an eye-level target positioned on the wall ~2 m in front of them, while in the EC condition the participants wore opaque swimming goggles. The order of the six trials was randomized across participants. Each 45 s trial started with quiet stance lasting for 15 s without vibration, followed by 10 s with vibration, and finishing with 20 s without vibration (Figure 1). A trial was repeated if participants lost balance or moved their hands from the hips or stepped trying to recover it. Three-minute intervals between trials were given to rest and washout any vibration effects (Wierzbicka et al., 1998).

### Data Collection

Eleven spherical markers were attached to the participant's skin on the seventh cervical vertebrae and, bilaterally, on the temples, the great trochanter, the femoral condyle, the calcaneus, and the proximal phalange of the first toe (hallux). The 3D-trajectories of these markers were registered at 250 Hz using a six-camera Smart-D BTS® motion capture system (Smart-D BTS, BTS Bioengineering S.p.A., Milan, Italy). A viewing space of 2.01 ± 0.28 m (anterior-posterior direction) × 2.25 ± 0.11 m (vertical direction) × 3.67 ± 0.41 m (medio-lateral direction) was calibrated before the data collection with a mean error of 0.338 ± 0.040 mm. Reconstruction of the 3D markers was done using the software Smart Tracker (BTS Bioengineering S.p.A., Milan, Italy).

### PCA Data Analyses, Kinematics in Posture Space, and Variables

The PManalyzer software (Haid et al., 2019) implemented in Matlab® (The MathWorks Inc., Natick, MA, USA) was used to fill gaps in the markers trajectories, normalize the kinematic data, and compute a PCA to obtain kinematic variables in posture space. Following, we briefly explain these three processes. The marker trajectories of each trial were checked and a PCA-based reconstruction algorithm (Federolf et al., 2013; Gløersen and Federolf, 2016) was used to fill gaps when it was necessary.

Posture analysis using PCA was utilized to identify linear whole-body movement patterns constituting the changes in posture that are observed during the analyzed time. Application of PCA to identify movement patterns within a sample requires to conduct a normalization process to account for anthropometrical differences between participants (Federolf et al., 2013; Federolf, 2016; Zago et al., 2017; Promsri et al., 2018; Haid et al., 2019). In the current study, the data of each participant were normalized by (1) subtracting the individual's mean posture vector (Federolf et al., 2013), (2) dividing by their mean Euclidian distance (Federolf et al., 2013), and (3) weighting each marker with its representative relative mass (Federolf, 2016; Gløersen et al., 2018). Weight factors applied to markers were calculated dividing the relative weight of each segment (de Leva, 1996) by the number of markers attached on the segment and, in case of markers placed on joints, masses of both segments were added (Federolf, 2016). Finally, one input matrix for the PCA was created by concatenating the normalized data of all participants.

The marker coordinates of the input matrix at a given time represented a posture vector. Applying PCA on these posture vectors quantified the postural variability via eigenvectors (PC_k_, where k indicates the order of the eigenvector), scores [PP_*k*_(t)], and eigenvalues EV_*k*_ (Troje, 2002; Daffertshofer et al., 2004; Verrel et al., 2009; Bockemühl et al., 2010). The PC_*k*_ form an orthonormal basis for a coordinate system in which the movements of the volunteers can be quantitatively compared. Each PC_*k*_ defines a specific pattern of how the markers move and thus how the body segments represented by the markers move. The temporal information on the postural position of a volunteer is quantified by the scores PP_*k*_(t), which are obtained by projecting each participant's normalized data onto the PC_*k*_. We interpret the PC_*k*_ and the PP_*k*_(t) as a new set of kinematic variables, called principal movements (PM_*k*_) (Federolf et al., 2012), where the PC_*k*_ defines the variable and the PP_*k*_(t) describe the evolution of the position in posture space over time (Federolf, 2016; Haid et al., 2018, 2019). Animated stick figures of the PM_*k*_ were created to facilitate an interpretation of what postural action each PM represents (Federolf et al., 2012, 2013; Federolf, 2016). Typically, the eigenvalues (EV_*k*_) are used to quantify the amount of postural variance associated with each PM_*k*_ (Federolf et al., 2013). However, Haid et al. (2018) proposed to use relative standard deviation, rSTD_*k*_, to quantify each PM_*k*_'s contribution to the volunteer's overall postural variations, since rSTD scales directly to the postural movement amplitude and not to its square. Group differences in the PP-rSTD_*k*_ were interpreted as differences in the composition of the postural movements (i.e., postural coordinative structure).

Individual postural coordinative structure changes during the trials can be analyzed and compared to other trials in different conditions or groups after the coordinate transformation into posture space (Federolf, 2016). Furthermore, by calculating the first and the second time-derivatives of PP_*k*_(t) one obtains principal velocities, PV_*k*_(t), and principal accelerations, PA_*k*_(t), respectively (Federolf, 2016). Interest in PV_*k*_ and PA_*k*_ during static balance relies on their relation to the sensory integration state and on the information they provide on how postural movements are controlled, respectively. Sensory receptors such as proprioception and vision are thought to be velocity sensitive, i.e., sensory information related to posture mainly favor rate information rather than absolute position information (Jeka et al., 2004). PV_*k*_ quantify the rate of postural coordinative structure changes. The relative contribution of each PV_*k*_ (relative standard deviation of the PV_*k*_, PV-rSTD_*k*_) thus characterizes how much rate-dependent sensory information each PM_*k*_ contributes. The PA_*k*_(t) represent the acceleration of the postural movement components. In quiet stance experiments it can be assumed that only gravity (which is constant) and muscle actions produce changes in the postural coordinative structure. Therefore, the PA_*k*_ can be used to characterize the neuromuscular control of the postural movements. We characterize the PA_*k*_ -time series via their relative standard deviation (PA-rSTD_*k*_) (Federolf, 2016; Haid et al., 2018; Promsri et al., 2018). The PA-rSTD_*k*_ inform about the relative contribution of each postural movement component to the overall postural accelerations. An altered PA-rSTD_*k*_, for example between trials, would suggest a change in the significance of the associated movement component for postural control (Longo et al., 2019). Noise amplification related to the differentiation process was reduced applying a Butterworth low-pass filter (order: 3th; cut off frequency: 7 Hz) (Promsri et al., 2018) before calculating PV_*k*_ and PA_*k*_.

All variables (PP-rSTD_*k*_, PV-rSTD_*k*_, and PA-rSTD_*k*_) were generated after dividing the PM_*k*_, PP_*k*_, PV_*k*_, and PA_*k*_ of each trial in four 10-s phases: (1) before vibration phase (BV), 5–15 s of the trial; (2) during vibration phase (DV), 16–25 s of the trial; (3) after vibration early phase (AV1), 26–35 s of the trial; and (4) after vibration late phase (AV2), 36–45 s of the trial. Variables were calculated for the first 6 PMs, in each participant, each trial, and each phase.

### Statistical Analysis

Since three trials were collected per condition, as a first step, trial effects were assessed over the two conditions (EO and EC) in each group with one-way analyses of variance (ANOVAs) with repeated measures in each dependent variable. The absence of significant trial effects allowed us to calculate the individual mean from the three standing trials for each variable and condition.

Shapiro-Wilk tests were conducted to check normality distribution and, when the normality test failed, data were transformed computing their square root and if the transformed data remained non-normal distributed then they were converted to ranks. The main goal of this study was analyzed using 4 (Group) × 4 (Phase) × 2 (Visual condition) ANOVAs with group (C-G, C-NG, A-G, and A-NG) as between-participant factor and phase (BV, DV, AV1, and AV2) and visual condition (EO and EC) as within-participants factors. Sphericity-corrected values by Greenhouse-Geisser were reported when appropriate. Pairwise comparisons and planned comparisons with Bonferroni correction were used to establish differences between the four groups, phases, and conditions, or to contrast group and phases effects across visual conditions.

The effect size was measured for the ANOVAs using η*p*^2^ (≥0.01: small effect; ≥0.06: medium effect; and ≥0.14: large effect). Statistical significance was set at *p* < 0.05 for all analyses. Only statistically significant results were reported. All statistical tests were performed with SPSS PASW Statistics 18 software (SPSS, Inc., Chicago, IL).

## Results

### Characterization of the First 6 Principal Postural Movements

The first six PMs explained 98.4 ± 1.7% of the overall postural variance across the four groups (C-G, C-NG, A-G, and A-NG), phases and conditions. Animated stick figures visualizing the first six PMs (Supplementary Video 1) showed qualitative differences among the PMs. Following, we characterize each PM with its associated explained variance. PM_1_ captured the anterior-posterior sway around the ankle joint (AP ankle strategy, 67.5 ± 16.9%); PM_2_ showed the medio-lateral sway around the ankle joint (ML ankle strategy, 14.9 ± 11.0%); PM_3_ represented flexion/extension movements about the hip (AP hip strategy, 11.0 ± 9.5%); PM_4_ captured trunk rotations in the transverse plane without head movements (trunk rotational strategy, 2.6 ± 2.5%); PM_5_ captured trunk rotations in the transverse plane together with head rotations in the same plane but in opposite direction (opposite trunk and head rotational strategy, 1.4 ± 1.4%); and PM_6_ showed flexion/extension movements of the knee (AP knee strategy, 1.0 ± 2.3%).

### Relative Contribution of Postural Movements

**Figure 2:**
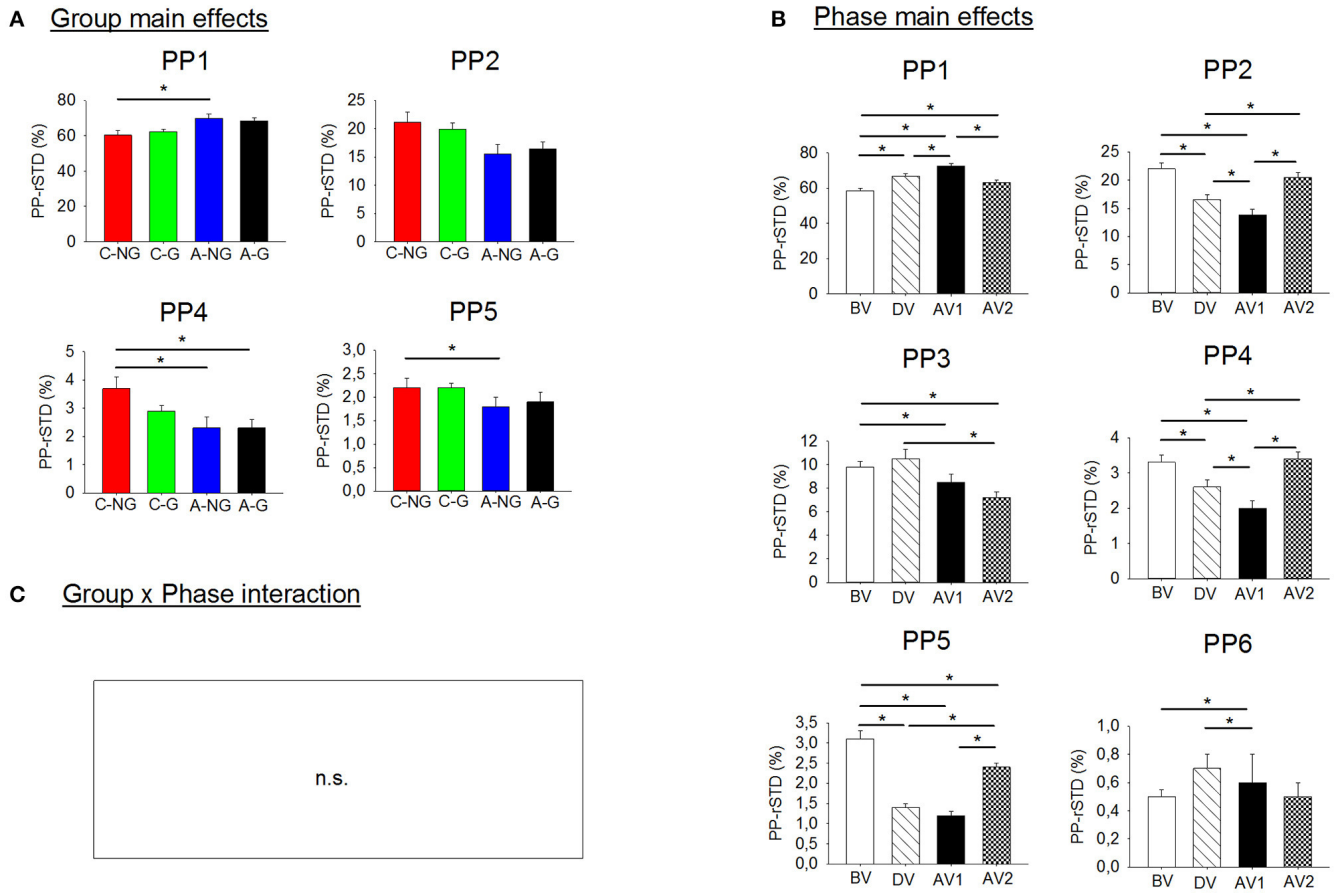
**(A)** Significant group main effects in the PPs (principal postural movement components). Means and standard deviations were plotted by group (children non-gymnasts, C-NG; children gymnasts, C-G; adults non-gymnasts, A-NG; and adults gymnasts, A-G); **(B)** significant phase main effects in the PP. Means and standard deviations were plotted by phase (BV, before vibration; DV, during vibration; AV1, after vibration early phase; AV2, after vibration late phase); and **(C)** group x phase interactions were not significant (n.s.). Asterisks (*) indicate significant difference in the *post-hoc* analyses.

Results from ANOVAs showed significant group main effects in the postural configuration in the PP-rSTD_1_, the PP-rSTD_2_, the PP-rSTD_4_, and the PP-rSTD_5_ (Table 2, Figure 2). *Post-hoc* results indicated that A-NG used the AP ankle strategy (PP-rSTD_1_) more than C-NG (*p* = 0.048), while trunk rotational strategies without or with head movements (PP-rSTD_4_ and PP-rSTD_5_, respectively) were performed to a greater extent by C-NG in comparison to A-NG (*p* = 0.025 and *p* = 0.024). However, no differences among groups in the *post-hoc* analysis for PP-rSTD_2_ were found. In addition, ANOVA results indicated significant phase main effects in all PP-rSTD components. In general and in contrast to BV, principal components related to movements in the AP direction for the ankle and knee (PP-rSTD_1_ and PP-rSTD_6_) presented a significant increase of the PP-rSTD values in DV and AV1 (Table 2, Figure 2), while movements in ML direction (PP-rSTD_2_) or rotations (PP-rSTD_4_ and PP-rSTD_5_) decreased their values during the DV and AV1. In addition, PP-rSTD_3_ showed a decrease in values for AV1 and AV2 compared to BV and DV.

**Table 2:** Significant results from the four (Group) x four (Phase) x two (Visual condition) ANOVAs in the principal postural movement components (PP).

	**ANOVA**						
**Variables**	**Main Effect or Interaction**	***F***	***df***	***p***	***η^2^p***	**Power**	***Post-hocs***
PP-rSDT_1_	Group	4.582	3, 73	0.005	0.158	0.87	C-NG < A-NG
	Phase	29.931	3, 73	<0.001	0.291	1.00	BV < DV, AV2 < AV1
	Visual condition	41.841	1, 75	<0.001	0.364	1.00	EO < EC
${\text{PP-rSDT}}_{2}^{\text{a}}$	Group	3.965	3, 73	0.011	0.140	0.81	–
	Phase	30.766	3, 73	<0.001	0.296	1.00	BV, AV2 > DV > AV1
	Visual condition	35.136	1, 75	<0.001	0.325	1.00	EO > EC
${\text{PP-rSDT}}_{3}^{\text{b}}$	Phase	8.136	3, 73	<0.001	0.100	0.99	BV > AV1, AV2; DV > AV2
	Visual condition	26.661	1, 75	<0.001	0.268	0.99	EO > EC
${\text{PP-rSDT}}_{4}^{\text{b}}$	Group	4.487	3, 73	0.006	0.156	0.86	C-NG > A-NG, A-G
	Phase	26.649	3, 73	<0.001	0.267	1.00	BV, AV2 > DV > AV1
	Visual condition	19.467	1, 75	<0.001	0.211	0.99	EO > EC
${\text{PP-rSDT}}_{5}^{\text{b}}$	Group	3.647	3, 73	0.016	0.130	0.78	C-NG > A-NG
	Phase	72.412	3, 73	<0.001	0.498	1.00	BV > AV2 > DV, AV1
	Visual condition	22.094	1, 75	<0.001	0.232	1.00	EO > EC
${\text{PP-rSDT}}_{6}^{\text{b}}$	Phase	4.980	3, 73	0.002	0.064	0.87	BV, DV < AV1
	Visual condition	7.395	1, 75	0.008	0.092	0.77	EO > EC

*C-NG, children non-gymnast; C-G, children gymnast; A-NG, adult non-gymnast; A-G, adult gymnast; EO, eyes open; EC, eyes closed; BV, before vibration phase; DV, during vibration phase; AV1, early after vibration phase; AV2, later after vibration phase; PP-rSDT, principal position relative standard deviation*.

a *Test of normality failed. Square root values were used to compute ANOVA*.

b *Test of normality failed. Values were transformed on ranks to compute ANOVA*.

**Figure 3:**
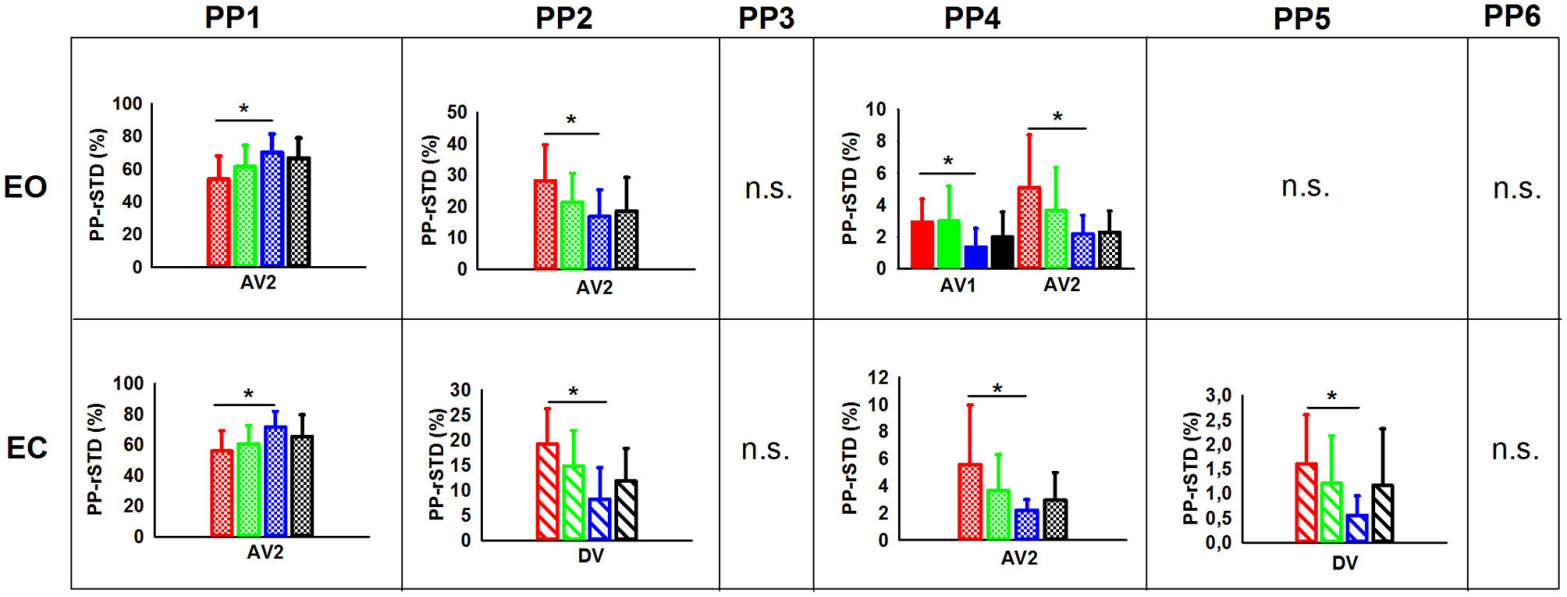
PP (principal postural movement components) means and standard deviations for the children [non-gymnasts (C-NG) in red and gymnasts (C-G) in green] and adults [non-gymnasts (A-NG) in blue and gymnasts (A-G) in black] by visual condition (EO, eyes open; EC, eyes closed). Phases were shaded differently (BV, before vibration; DV, during vibration; AV1, after vibration early phase; AV2, after vibration late phase), but only phases with significant differences between groups were plotted. Asterisks (*) indicates significant differences from planned comparisons; n.s. indicates non-significant results.

The planned comparisons exposed that in the EO condition (Figure 3) the C-NG group presented significantly higher PP-rSTD_4_ values than A-NG during the after vibration early phase (AV1) (*p* = 0.025), while in the after vibration late phase (AV2) C-NG compared to A-NG showed a decreased use of the AP ankle strategy (*p* = 0.016) but an increased use of the ML ankle strategy (PP-rSTD_2_) and the trunk rotational strategy (PP-rSTD_4_) (*p* = 0.039 and *p* = 0.043, respectively). Differences were also found in the planned comparisons conducted in the EC condition (Figure 3). Children non-gymnasts exhibited larger values of PP-rSTD_2_ (*p* = 0.001) and PP-rSTD_5_ (*p* = 0.010) than adults with no gymnastic experience during vibration (DV phase). In the AV2, C-NG used less the AP ankle strategy (PP-rSTD_1_, *p* = 0.020) and more the trunk rotational strategy (PP-rSTD_4_, *p* = 0.050) than A-NG.

### Speed of Postural Movements

**Figure 4:**
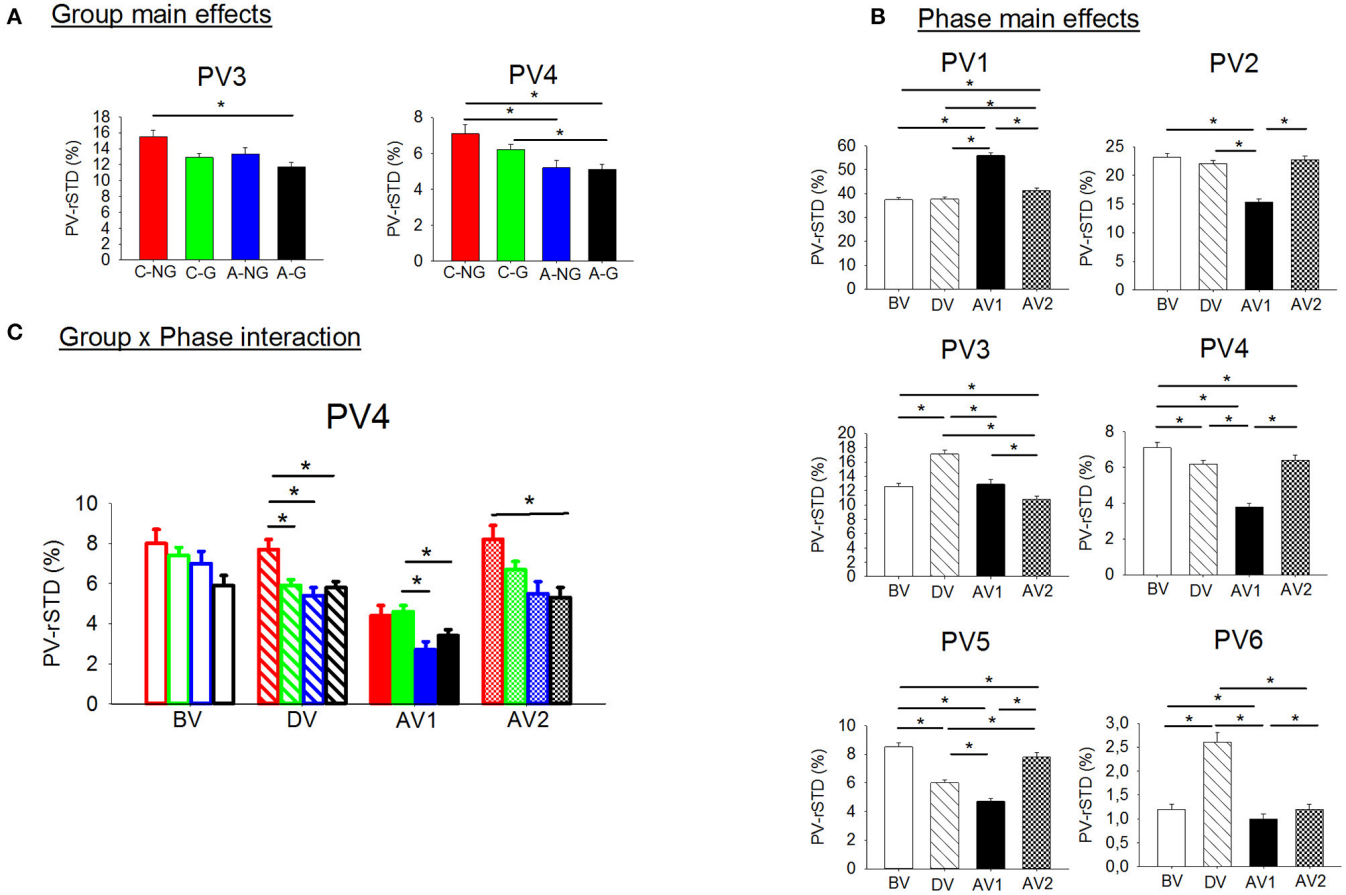
**(A)** Significant group main effects in the PVs (principal velocities). Means and standard deviations were plotted by group (children non-gymnasts, C-NG, children gymnasts, C-G, adults non-gymnasts, A-NG, and adults gymnasts, A-G); **(B)** significant phase main effects in the PV. Means and standard deviations values were plotted by phase (BV, before vibration; DV, during vibration; AV1, after vibration early phase; AV2, after vibration late phase); and **(C)** significant group x phase interactions in the PVs. (*) indicates significant differences in the *post-hoc* analyses.

Significant group and phase main effects and group x phase interactions were found by ANOVAs for the sensory information contribution variables (Table 3, Figure 4). Follow up *post-hocs* on the PV-rSTD_4_ group main effect showed that children groups (C-NG and C-G) exhibited larger velocity rotating their trunk without head movements (PV-rSTD_4_) than their respective gymnastic experience adult groups (*p* = 0.017 and *p* = 0.050 for NG and G, respectively). In addition, C-NG showed higher values in PV-rSTD_3_ and PV-rSTD_4_ than the A-G (*p* = 0.005 and *p* = 0.005, respectively). Phase main effects showed that compared to BV, velocity increased during AV1 for the PV-rSTD_1_ and during DV for the PV-rSTD_3_ and PV-rSTD_6_. On the other hand, the velocity decreased particularly in AV1 for the PV-rSTD_2_, PV-rSTD_4_, and PV-rSTD_5_ compared to BV. Pairwise comparisons from the Group x Phase interaction revealed differences among groups in PV-rSTD_4_ for all phases except in the before vibration phase (BV). Children without gymnastic experience showed higher values of PV-rSTD_4_ than C-G (*p* = 0.026), A-NG (*p* = 0.005), and A-G (*p* = 0.022) during DV. In the early phase after vibration (AV1) C-G presented larger velocity than A-NG (*p* = 0.001) and A-G (*p* = 0.010) while in the after vibration late phase (AV2) C-NG showed larger trunk rotation velocity than A-G (*p* = 0.008).

**Table 3:** Significant results from the four (Group) x four (Phase) x two (Visual condition) ANOVAs in the principal velocities (PV).

	**ANOVA**						
**Variables**	**Main effect or Interaction**	***F***	***df***	***p***	***η^2^p***	**Power**	***Post-hocs***
${\text{PV-rSDT}}_{1}^{\text{a}}$	Phase	118.798	3, 73	<0.001	0.619	1.00	BV, DV < AV2 < AV1
	Visual condition	349.413	1, 75	<0.001	0.827	1.00	EO < EC
PV-rSDT_2_	Phase	51.210	3, 73	<0.001	0.412	1.00	BV, DV, AV2 > AV1
	Visual condition	40.059	1, 75	<0.001	0.834	1.00	EO > EC
${\text{PV-rSDT}}_{3}^{\text{a}}$	Group	4.088	3, 73	0.010	0.144	0.83	C-NG > A-G
	Phase	37.573	3, 73	<0.001	0.340	1.00	AV2 < BV, AV1 < DV
	Visual condition	55.816	1, 75	<0.001	0.433	1.00	EO > EC
${\text{PV-rSDT}}_{4}^{\text{a}}$	Group x Phase	2.770	9, 219	0.004	0.102	0.95	DV: C-NG > C-G, A-NG, A-G AV1: C-G > A-NG, A-G AV2: C-NG > A-G
	Group	5.779	3, 73	0.001	0.192	0.94	C-NG > A-NG, A-G; C-G > A-G
	Phase	92.880	3, 73	<0.001	0.560	1.00	BV > DV, AV2 > AV1
	Visual condition	124.580	1, 75	<0.001	0.631	1.00	EO > EC
${\text{PV-rSDT}}_{5}^{\text{a}}$	Phase	129.545	3, 73	<0.001	0.640	1.00	BV > AV2 > DV > AV1
	Visual condition	152.758	1, 75	<0.001	0.677	1.00	EO > EC
${\text{PV-rSDT}}_{6}^{\text{b}}$	Phase	81.162	3, 73	<0.001	0.526	1.00	AV1 < BV, AV2 < DV
	Visual condition	77.165	1, 75	<0.001	0.514	1.00	EO > EC

*C-NG, children non-gymnast; C-G, children gymnast; A-NG, adult non-gymnast; A-G, adult gymnast; EO, eyes open; EC, eyes closed; BV, before vibration phase; DV, during vibration phase; AV1, early after vibration phase; AV2, later after vibration phase; PV-rSDT, principal velocity relative standard deviation*.

a *Test of normality failed. Square root values were used to compute ANOVA*.

b *Test of normality failed. Values were transformed on ranks to compute ANOVA*.

**Figure 5:**
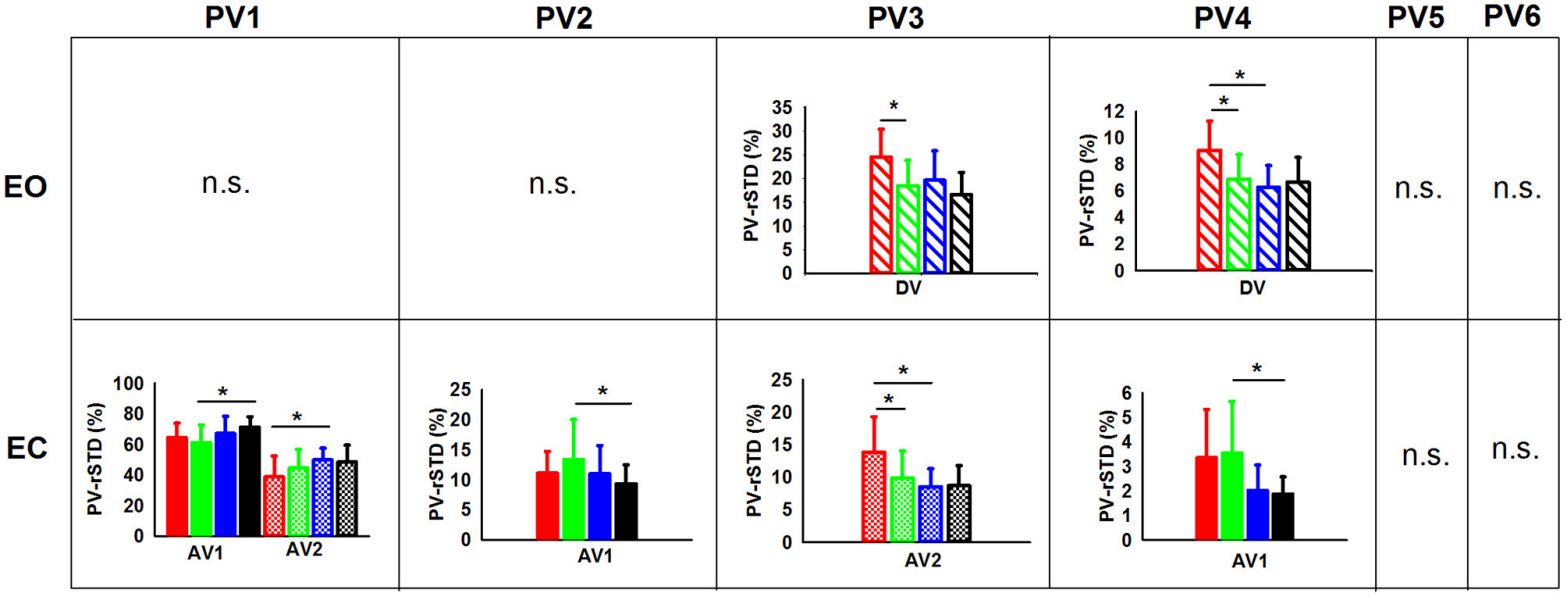
PVs (principal velocities) means and standard deviations for the children [non-gymnasts (C-NG) in red and gymnasts (C-G) in green] and adults [non-gymnasts (A-NG) in blue and gymnasts (A-G) in black] by visual condition (EO, eyes open; EC, eyes closed). Only phases (BV, before vibration; DV, during vibration; AV1, after vibration early phase; AV2, after vibration late phase) with significant differences between groups were plotted (phases were shaded differently). (*) indicates significant differences from planned comparisons. (n.s.) indicates non-significant results.

In addition, the planned comparisons conducted in the EO and the EC conditions showed differences between groups in several PV-rSTD variables (Figure 5), but EO only during vibration while EC only during after vibration. Children non-gymnast with EO during DV exhibited larger values of AP hip flexion/extension velocity (PV-rSTD_3_) than C-G (*p* = 0.022) and larger values of the trunk rotation velocity (PV-rSTD_4_) than C-G (*p* = 0.017) and A-NG (*p* = 0.006). The EC trials revealed differences between C-G and A-G only during AV1. That is, C-G showed smaller velocity for the AP ankle movements (PV-rSTD_1_) (*p* = 0.003) and larger velocity for the ML ankle movements (PV-rSTD_2_) (*p* = 0.037) and trunk rotations without head velocity (PV-rSTD_4_) (*p* = 0.001). On the other hand, C-NG participants in the EC condition showed group differences only during AV2. That is, C-NG had lower PV-rSTD_1_ values than A-NG (*p* = 0.050) but larger PV-rSTD_3_ values than C-G (*p* = 0.045) and A-NG (*p* = 0.014).

### Acceleration of Postural Movements

**Figure 6:**
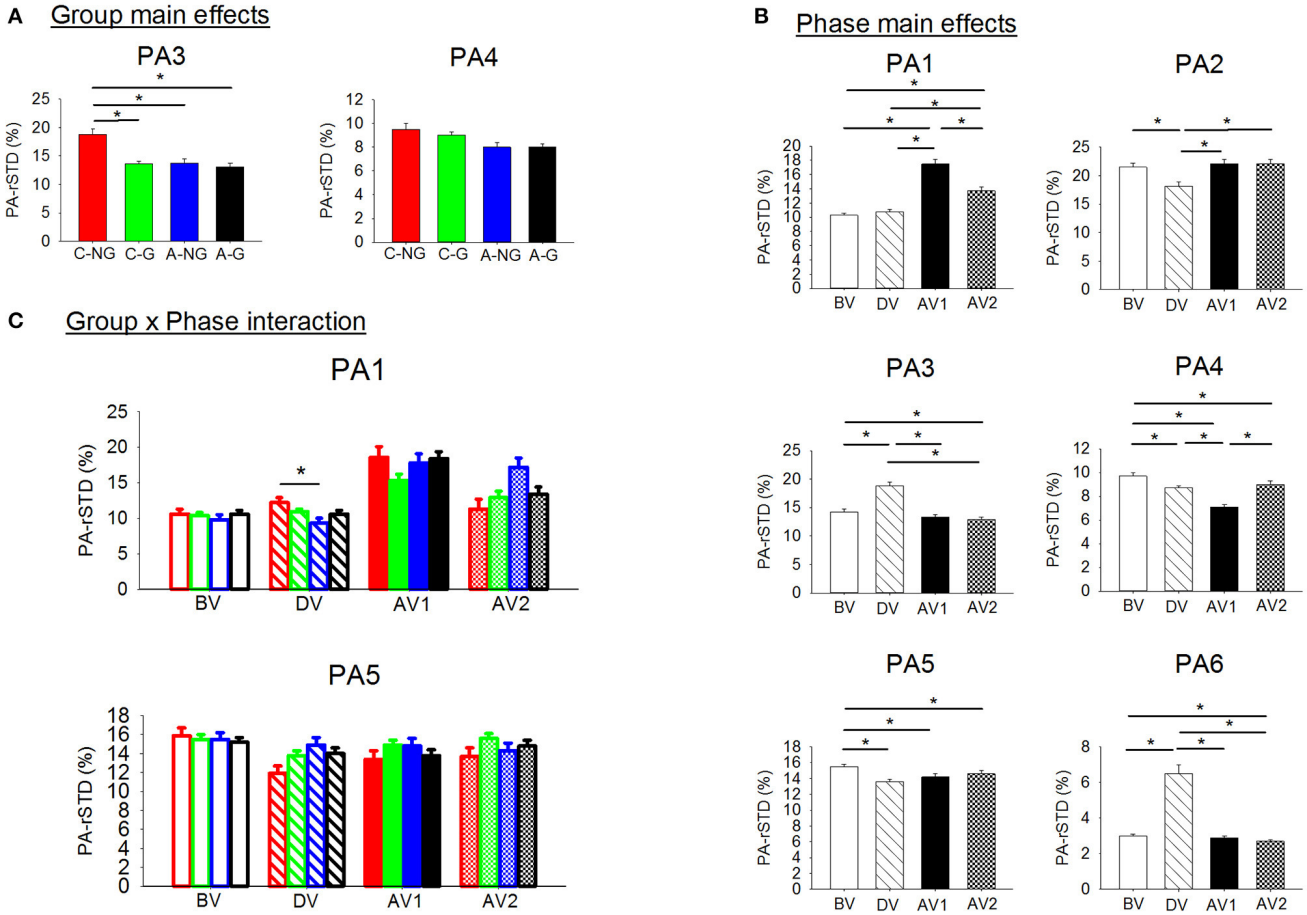
**(A)** Significant group main effects in the PAs (principal acceleration). Means and standard deviations were plotted by group (children non-gymnasts, C-NG, children gymnasts, C-G, adults non-gymnasts, A-NG, and adults gymnasts, A-G); **(B)** significant Phase main effects in the PA. Means and standard deviations values were plotted by phase (BV, before vibration; DV, during vibration; AV1, after vibration early phase; AV2, after vibration late phase); and **(C)** significant group x phase interactions in the PAs. (*) indicates significant differences in the *post-hoc* analyses.

The ANOVAs results for the variables that quantified the magnitude of the neuromuscular intervention (PA-rSTD) showed a significant group main effect on AP hip strategy (PA-rSTD_3_) and trunk rotation without head movements (PA-rSTD_4_), phase main effects in all PA-rSTD components, and a significant group x phase interaction on the AP ankle strategy (PA-rSTD_1_) and the trunk rotation with head movements (PA-rSTD_5_) (Table 4, Figure 6). Larger values of hip flexion/extension acceleration (PA-rSTD_3_) were found in the C-NG compared to C-G (*p* = 0.001), A-NG (*p* = 0.002), and A-G (*p* = 0.001). However, no *post-hoc* differences among groups in thePA-rSTD_4_ were found. Phase main effects showed that larger acceleration occurred during AV1 for the PA-rSTD_1_ and during DV for the PA-rSTD_3_ and PA-rSTD_6_ compared to BV. On the other hand, and compared to BV, the acceleration presented lower values in DV for the PA-rSTD_2_, PA-rSTD_4_, and PA-rSTD_5_, and during the AV1 there were also lower values for the PA-rSTD_4_ and PA-rSTD_5_. Pairwise comparisons from the group x phase interaction only revealed that C-NG moved the ankle in the AP direction with larger acceleration than A-NG (*p* = 0.020) in DV.

**Table 4:** Significant results from the four (Group) x four (Phase) x two (Visual condition) ANOVAs in the principal accelerations (PA).

	**ANOVA**						
**Variables**	**Main Effect or Interaction**	***F***	***df***	***p***	***η^2^p***	**Power**	***Post-hocs***
${\text{PA-rSDT}}_{1}^{\text{b}}$	Group x Phase	3.712	9, 219	<0.001	0.132	0.99	DV: C-NG > A-NG
	Phase	70.261	3, 73	<0.001	0.490	1.00	BV, DV < AV2 < AV1
	Visual condition	103.644	1, 75	<0.001	0.587	1.00	EO < EC
${\text{PA-rSDT}}_{2}^{\text{a}}$	Phase	20.664	3, 73	<0.001	0.221	1.00	BV, AV1, AV2 > DV
	Visual condition	5.772	1, 75	0.019	0.073	0.66	EO > EC
${\text{PA-rSDT}}_{3}^{\text{a}}$	Group	8.090	3, 73	<0.001	0.250	0.99	C-NG > C-G, A-NG, A-G
	Phase	42.095	3, 73	<0.001	0.366	1.00	DV > BV, AV1, AV2; BV > AV2
${\text{PA-rSDT}}_{4}^{\text{a}}$	Group	3.846	3, 73	0.013	0.136	0.80	-
	Phase	36.821	3, 73	<0.001	0.335	1.00	BV > DV, AV2 > AV1
	Visual condition	23.368	1, 75	<0.001	0.242	1.00	EO > EC
	Group x Phase	2.510	9,219	0.009	0.094	0.929	-
	Phase	13.631	3, 73	<0.001	0.157	1.00	BV > DV, AV1, AV2
PA-rSDT_5_	Visual condition	27.816	1, 75	<0.001	0.276	1.00	EO > EC
PA-rSDT_6_^b^	Phase	118.791	3, 73	<0.001	0.619	1.00	AV2 < BV < DV; AV1 < DV

*C-NG, children non-gymnast; C-G, children gymnast; A-NG, adult non-gymnast; A-G, adult gymnast; EO, eyes open; EC, eyes closed; BV, before vibration phase; DV, during vibration phase; AV1, early after vibration phase; AV2, later after vibration phase; PA-rSDT, principal acceleration relative standard deviation*.

a *Test of normality failed. Square root values were used to compute ANOVA*.

b *Test of normality failed. Values were transformed on ranks to compute ANOVA*.

**Figure 7:**
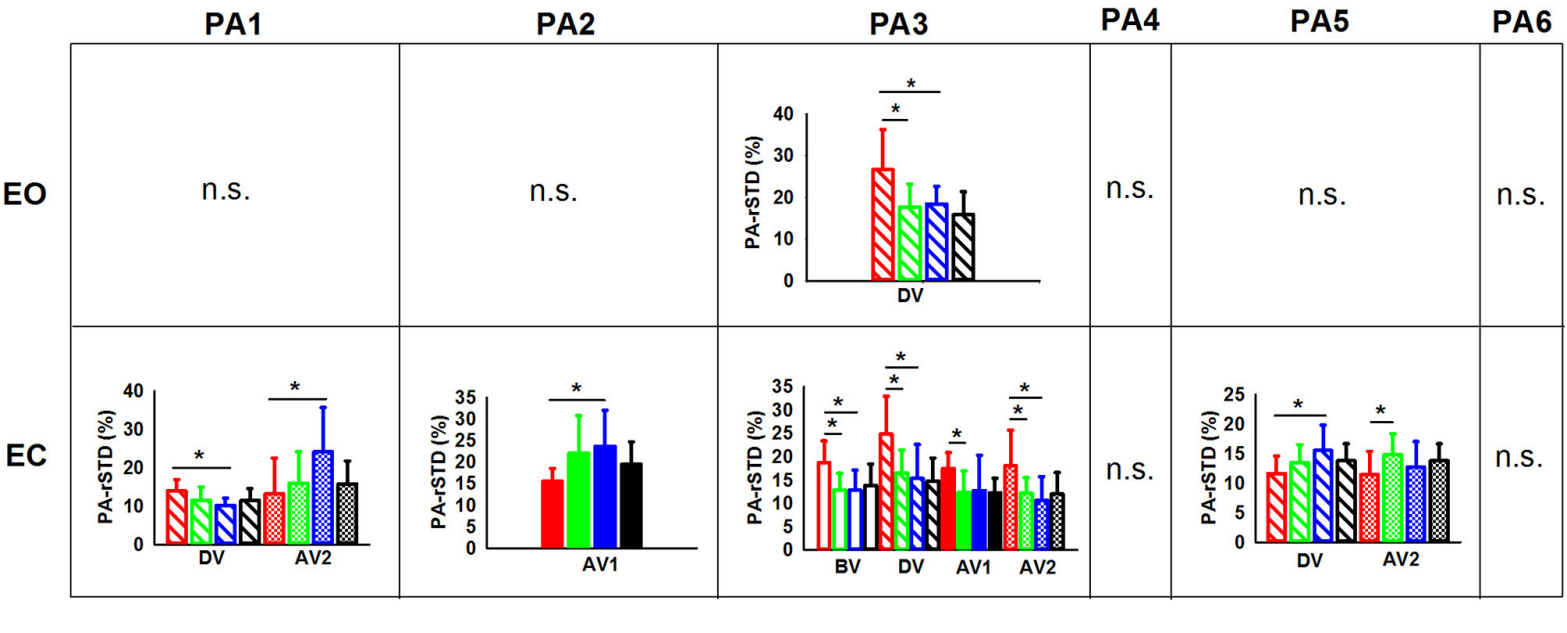
PAs (principal velocities) means and standard deviations for the children [non-gymnasts (C-NG) in red and gymnasts (C-G) in green] and adults [non-gymnasts (A-NG) in blue and gymnasts (A-G) in black] by visual condition (EO, eyes open; EC, eyes closed). Only phases (BV, before vibration; DV, during vibration; AV1, after vibration early phase; AV2, after vibration late phase) with significant differences between groups were plotted, with different shading used for each phase. (*) Indicates significant differences from planned comparisons. (n.s.) Indicates non-significant differences.

Planned comparisons (Figure 7) in the EO trials only revealed C-NG showing larger values of PA-rSTD_3_ in comparison to C-G (*p* = 0.002) and A-NG (*p* = 0.033) during DV. On the other hand, planned comparisons in the EC condition showed significant differences among groups in all four phases analyzed. Children without gymnastic experience presented larger acceleration of the AP hip movements than C-G (*p* = 0.003) and A-NG (*p* = 0.012) during BV. Similarly, C-NG showed higher values of PA-rSTD_3_ than the other two mentioned groups in the other phases: DV (C-NG vs. C-G: *p* = 0.003; C-NG vs. A-NG: *p* = 0.003), AV1 (C-NG vs. C-G: *p* = 0.015), and AV2 (C-NG vs. C-G: *p* = 0.014; C-NG vs. A-NG: *p* = 0.002). In addition, planned comparisons in DV revealed larger values of PA-rSTD1 (*p* = 0.022) for C-NG than A-NG, while PA-rSTD_5_ values in C-NG were smaller in comparison to A-NG (*p* = 0.028). Values of the ML ankle accelerations (PA-rSTD_2_) were smaller for the C-NG than A-NG (*p* = 0.039) during AV1. Focusing in the after vibration late phase (AV2), C-NG showed smaller values of PA-rSTD_1_ than A-NG (*p* = 0.023) and of PA-rSTD_5_ compared to C-G (*p* = 0.048).

## Discussion

### Main Results

This research was designed to better understand changes in the postural movements performance and control during bipedal standing in different proprioceptive and visual conditions subjected to sensory reweighting processes and how these changes were affected by age and gymnastic experience. Our results showed that sensory information alteration led to changes in the postural coordinative structure (PP), sensory integration state (PV), and control (PA), especially when individuals were under the most demanding condition (i.e., EC and vibration), and also provided evidence that these changes did not disappear when the vibration stopped (confirming Hypothesis 1). Regarding the age effect, differences in the coordinative structure, sensory state and motor control variables were found across age but mostly between both non-gymnastic groups and some of these differences were found 10–20 s after vibration (partially confirming Hypothesis 2). In addition, gymnastic experienced children exhibited an adult-like postural coordinative structure, sensory state, and control during the reweighting processes while no differences due to gymnastic practice were found between the adult groups (partially confirming Hypothesis 3).

### Characterization of the First 6 Principal Postural Movements

The current findings showed that participants' movements during the bipedal stance trials were predominantly performed by the ankle and hip in the anterior-posterior direction (AP) (i.e., PM_1_ and PM_3_, respectively). The so-called ankle and hip strategies mentioned in the literature, together, accounted approximately for an 80% of the movement variance. On the other hand, ankle movements in the medio-lateral direction (ML) (i.e., PM_2_) represented 15% of the movement variance while the rest of the variance (i.e., 5%) was explained by trunk rotations (without or with head movements) (i.e., PM_4_ and PM_5_) and knee flexion-extension movements (i.e., PM_6_). These findings support studies suggesting that the ankle and hip strategies do not fully explain the postural control movement during bipedal stance and other multi-segment movements should be considered (Hsu et al., 2007; Pinter et al., 2008). The postural movements, and their corresponding percentages of explained variance, obtained in the current study were similar to those reported in adult bipedal posture studies also using data-driven PCA (Federolf et al., 2013; Federolf, 2016) with the exception of breathing movements as part of the principal components. It is known that the number of markers distributed over the subject influences the results (Federolf et al., 2014). The biomechanical model used in our study (without a chest marker) did not allow collecting information about possible breathing movements but seemed to provide enough information about the multi-joint postural movements and the PCA methodology appeared to have sufficient sensitivity to recognize them.

### Postural Coordinative Structure

Independent of the vision condition, the interplay among postural movements (i.e., coordinative structure of the posture) changed across phases when participants' sensory reweighting capability was tested manipulating proprioception (i.e., vibration). Participants during the vibration phase (when proprioception was perturbed) increased the use of ankle and knee AP movements compared to baseline while ankle movements in the ML and trunk rotations (without or with head movements) were decreased. These changes in the postural coordinative structure remained during the first 10 s after vibration was removed (i.e., after vibration early phase,), a period when participants had to reweight the proprioception information. However, between 10 and 20 s after vibration was stopped (i.e., after vibration later phase), participants showed postural movements values similar to those found before the vibration (i.e., before vibration phase), except for the ankle movements in the AP which still moved more than before the proprioceptive manipulation. These results suggested that the proprioceptive manipulation on the Achilles' tendons had larger effects on the ankle movements in the AP and on individuals' ability to return them to the initial state (i.e., stability) than the other postural movements (Eklund, 1972; Thompson et al., 2007; Teasdale et al., 2017). The increase in knee movements could be associated with an attempt to cooperate with the ankle in reducing and slowing down the center of mass (COM) movements in the AP in the during and early-after vibration phases (Alexandrov et al., 2005; Pinter et al., 2008; Reimann and Schöner, 2017). In contrast, the decreased trunk rotation movements during these same phases could be related to a decrease in postural control complexity (Haid and Federolf, 2018; Wachholz et al., 2019a; Promsri et al., 2020). The stabilization of the trunk rotation and knee movements during the after vibration later phase may indicate that their altered contributions were no longer needed to stabilize posture.

Differences in the postural coordinative structure were observed between children and adults without experience in gymnastics (i.e., age effect) while our results did not show any gymnastic experience effects between adults. Children without gymnastic experience in both visual conditions (i.e., EO and EC) exhibited less use of the ankle movements in AP and more use of the trunk rotations (without or with head movements) than the adults without gymnastic experience, mainly during the early and later phases after vibration. Additional differences between children and adults without gymnastic experience were revealed during vibration and eyes closed condition (i.e., the most demanding condition). Specifically, the ankle movements in the ML and the trunk rotations with head movements were used more in children without gymnastic experience, while no differences were found with eyes open.

In the ecological dynamics approach, perceptual-action solutions are supported by the inherent degeneracy of the system (i.e., ability to use elements that are structurally different to perform the same function) (Edelman and Gally, 2001; Seifert et al., 2013; Mason, 2015). In our study, degeneracy could facilitate individuals' exploitation of their sensory reweight capacity to utilize different coordinative structures to stabilize posture when facing perturbations or recovering from them. It has been suggested that developmental level (van Geert, 1994; Edelman and Gally, 2001; Mason, 2010, 2015) and expertise in performing a task (Seifert et al., 2013; Barris et al., 2014; Mason et al., 2015; Busquets et al., 2016) could modify an individual perception-action solution. We propose that the children without gymnastic experience results were a consequence of an under-developed degeneracy system which diminished either the capability to re-integrate proprioceptive information to perform different coordinative structures, or the possibility of co-existing modes of coordinative structures (Edelman and Gally, 2001; Mason, 2010, 2015; Seifert et al., 2013; Mason et al., 2015). However, not all sensory modality manipulations had the same effects. Children without gymnastic experience were able to reweight visual information when proprioception was perturbed and performed postural movements similar to those of adult groups or children with gymnastic experience suggesting that this group relied more on visual information. Interestingly, the postural coordinative structures of the children with gymnastic experience were similar to those of adults suggesting that practicing gymnastics could promote degeneracy capacity closer to adult level when faced with this particular task.

### Postural Sensory Integration State

Postural movements can be seen as a consequence of the actions to control the system in a stance position (Kiemel et al., 2002; Peterka, 2002) but also as a reflection of a purposeful neural mechanism related to test limits of vertical posture stability (Riccio, 1993; Jeka et al., 2004; Mochizuki et al., 2006). It has been suggested that the movements' velocity is the most accurate sensory information used to stabilize posture during quite stance (Kiemel et al., 2002; Jeka et al., 2004). Therefore, it is expected that velocity of the postural movements (i.e., principal velocities, PV) would increase when a sensory source is perturbed and participants would use it to explore their stability limits in this new context. Independently of the visual condition, when proprioception was perturbed our results showed that the AP movements of the hip and knee increased their velocity during vibration while the ankle AP velocity maintained similar values to those before vibration. On the other hand, after vibration the ankle AP movement velocity increased while the hip and knee returned to initial before vibration values. Movements in other directions (ML and rotations) gradually diminished their velocity from the before vibration phase to the early after vibration phase and they recovered initial values later between 10 and 20 s after vibration.

Changes in the principal velocities during vibration could be interpreted as participants perceiving that proprioceptive information from ankle AP movements was not reliable to maintain the posture due to potentially conflicting sources of sensory information (Eklund and Hagbarth, 1966; Hayashi et al., 1981; McKay et al., 2014; Busquets et al., 2018), therefore they sought more information from supplementary joints in the AP direction (hips and knees). After vibration, participants returned to ankle proprioceptive information but with larger velocities during the early reweighting processes. These larger velocities after vibration could correspond to an exploratory approach to retune the stability limits defined by the ankle movements and also some possible after vibration effects. Abundance of proprioceptive sources distributed all over the body could have allowed participants to acquire useful information from other parts of the body when sources from the ankle were not reliable. Therefore, reweighting processes could involve changes in the relative use of several information types (e.g., increasing the use of proprioceptive information when eyes are closed) but also include changes in the relative use of the kinesthetic information from different body location (e.g., increasing the use of proprioceptive information from hips and knees when ankle information is altered). On the other hand, reduction of the velocities in the other directions (ML and rotations) could be attributed to an attempt to reduce information at minimum necessary to control movements but to allow the system to work in the main altered direction (i.e., AP direction). Theoretically, changes in the relevance of the sensory information to maintain perceptual stability (of the environment and the own body) during task performance could be explained by the existence of a perceptual structure configured by a high-dimensional space of afferent-efferent variables to percept the joint positions (i.e., iso-perceptual manifolds of the joints) (Latash, 2018b, 2020; Cuadra and Latash, 2019). Perturbation in the ankle proprioceptive sources could generate changes in the iso-perceptual manifold configuration (i.e., reweighting processes) that lead to create new perceptual structure (i.e., a non- perceptually equivalent structure) losing perception stability and producing changes also in the postural movements.

When age effect was explored in the PVs, children showed larger trunk rotation without the head movement velocity than the adults groups. Particularly, children without gymnastic experience were not able to contain the velocity of the trunk rotations without head movements during and after the vibration, while children with gymnastic experience failed to reduce this velocity during the first 10 s after vibration. Not reducing the trunk rotation velocities would add complexity to the perceptual structure used by children without gymnastic experience increasing the difficulty to accomplish an effective perceptual-action coupling. In addition, children without gymnastic experience in the EC condition presented lower values of ankle AP velocities and larger values of hip flexion-extension velocities than adults without gymnastic experience in the later after vibration phase. This result could be an indication that these children were not able at the end of the trial to return to initial perceptual structure and fully use the ankle information in the AP direction while relying more in the hip sensory information.

Differences because of gymnastic experience were also found between the children groups while the adults groups presented similar results. Children with gymnastic experience showed lower velocities in the trunk rotation without head movements during vibration and in the hip flexion-extension movements in the later phase after vibration. Gymnastic practice during childhood could impact the ability to change the perceptual structure diminishing the complexity of the sensory information constraining velocities of the trunk rotations and decreasing the necessity to use hip sensory information. Given that an existing or emerging perceptual structure has to be constrained by an a priori knowledge of the body and their relations with the environment, it could be argued that experience acquired by gymnastics practice (or age, based on the above results) changed the iso-perceptual manifolds of the joints or their relationship in the perceptual structures to better manage reweighting processes.

### Control of Postural Movements

Postural stability requires constant neuromuscular interventions (i.e., actions) to be coupled with perception. The principal accelerations (PA) are used to quantify the actions (“accelerations”) performed to maintain bipedal standing in different conditions (Federolf, 2016; Haid et al., 2018; Promsri et al., 2018; Wachholz et al., 2019b; Ó'Reilly and Federolf, 2021). Participants in our study showed that AP movements of the hip and knee increased their acceleration during vibration while ankle AP acceleration maintained similar values to those of before vibration. On the other hand, after vibration the ankle AP movement acceleration increased while the hip and knee returned to before vibration values. Movements in other directions (ML and rotations) diminished their accelerations during vibration. While ankle ML movement recovered their initial values early after vibration, trunk rotation movements recovered initial values later (between 10 and 20 s after vibration).

Given that larger muscular forces are necessary to stop or produce larger accelerations, larger muscular actions during vibration can be inferred from the increased hip and knee PA values. Interestingly, the acceleration values of the ankle AP movement did not change despite the vibration. Vibration generates perceptual effects about joint positions and velocities (Goodwin et al., 1972; Hayashi et al., 1981; Sittig et al., 1985; Proske and Gandevia, 2012) but also involves illusions of force (Feldman and Latash, 1982; Proske and Gandevia, 2012; Reschechtko et al., 2018) and induces the tonic vibration reflex (Eklund and Hagbarth, 1966; Wierzbicka et al., 1998). The challenging condition produced by the loss of reliability in the ankle proprioceptive information, the illusion of force production, and the tonic vibration reflex could increase muscle co-activation and consequently the ankle apparent stiffness. An increased co-activation that blocks ankle movements would lead to a loss of postural stability with faster movement changes of the COM. On the other hand, allowing movements, as participants did, could be a mechanism to stabilize posture at the same time that ensures abundance (i.e., availability of possible solutions) in poorly predictable contexts (i.e., perturbations) that require corrective actions (Latash, 2018a; Yamagata et al., 2019). In addition, to maintain the COM sway under control, the knee and hip movements became more compliant while increasing their movements and muscular actions to diminish the COM movements in a controlled range. After vibration the tonic vibration reflex disappeared but the perceptual structure had to be reweighted and coupled with the actions. Due to the possibility to rely again in the ankle movements to control the COM sway, the knee and hip accelerations returned to initial levels. However, posture stability was not achieved instantly (the COM movements may be in a controlled range but surely with more movements than usual) and therefore the ankle muscle actions increased (i.e., accelerations) to control posture and, at the end, stabilize the whole body.

Movements in directions less affected by vibration (ML and rotations) decreased their accelerations during vibration and recovered their initial values in the early after vibration phase (ankle ML movements) and in the later after vibration phase (trunk rotations without and with head movements). Participants appeared to avoid wasteful movements in directions less affected by vibration and in consequence they reduced their accelerations probably by muscle co-activations. These acceleration reductions could have been achieved by reducing non-essential movements in these directions. Theoretically, these results could occur because, to do any task, different perceptual-action solutions (i.e., motor equivalence) are developed with movements that lead to no changes in the performance (i.e., motor equivalent components) and movements that modify the performance (i.e., non-motor equivalent components) (Scholz et al., 2007; Mattos et al., 2011; Latash, 2018b). The configuration of the postural solutions (motor equivalences) found in our study might be governed by the “act on the most nimble” rule which is expected to be specific to problems of restoring stability of actions (Akulin et al., 2019; Yamagata et al., 2020). Following the “act on the most nimble” rule, changes in control coordinates occurred for the postural movement that shows the fastest deviation from its expected trajectory (i.e., ankle movements in the AP direction). Corrective action in ankle movements would not be enough to stabilize posture, and then the controller acts along the second fastest coordinate, and so on until posture is stabilized in the new context (when vibration appeared and then when vibration disappeared). In addition, reducing movements in the ML and rotational movements could allow individuals to obtain more reliable or better reference frame from the vestibular system to keep vertical posture (Mergner et al., 2009a,b; Héroux et al., 2015).

Children presented differences compared to adults in the control of their postural movements only when the comparison was done between groups without gymnastic experience. Non-gymnast children showed larger accelerations of the hip movements compared to the non-gymnast adults. Interestingly, in the EC condition this larger hip acceleration was found before the vibration application and consistently appeared during and after vibration phases, while in the EO condition these differences only were found during vibration. These results suggested that children without gymnastic experience were able to control posture similarly to adults but they relied on the visual information to achieve this adult like postural control. No-visual information could result in excessive increase in COM movements that led to increase the hip actions in the new perceptual-action solution in order to stabilize posture. In addition, the fact that even with the visual information available, children with no gymnastic experience were more affected by vibration can be interpreted as an undeveloped capacity to reweight properly sensory information and to elaborate new perceptual structure. In addition, ankle acceleration values in the AP direction of the non-gymnast children during vibration were higher than values found in non-gymnast adults. If we consider that their hip control was undeveloped, it could be suggested that children without gymnastic experience were not able to stabilize COM movements using hip actions and, in the absence of visual information, they continue using ankle actions even when proprioceptive information was not reliable. On the other hand, given the difficulty to control movements in the AP direction, children without gymnastic experience had the capacity to reduce accelerations in the trunk rotations with head movements during vibrations and in the ankle ML movements after vibration. These were directions less affected by vibration and children could decrease their accelerations in an attempt to prioritize AP movements control in their perceptual-action solutions.

Similar differences in the hip accelerations between non-gymnasts children and adults groups were also found between children with different gymnastic experience, with non-gymnast children showing an overall larger hip acceleration than gymnastic children. Taken together with the lack of differences between children and adults with gymnastic experience in any of the movement acceleration, it seems that gymnastic experience enhanced the development of the postural control during childhood.

### Limitations

The experimental set up used in the current study allowed to identify differences in the postural performance and control by age and gymnastic experience during reweighting processes, however, it has to be recognized that the biomechanical model and the kind of movements that can be measured and analyzed are limited by the number and the placements of reference markers. Nevertheless, the marker set employed in the current study did allow quantification of the main postural control movements produced (Horak et al., 1990; Runge et al., 1999). In addition, given that sensory and motor adjustments seem to be task-specific (Ringhof and Stein, 2018) and highly related to motor experience (Paillard, 2017), limited generalization could be applicable to other tasks and sports or forms of physical activity. Lastly, when interpreting PA variables to characterize neural control of postural movements, one needs to be aware that these accelerations are not only a product of active muscle forces, but may also stem from gravitational or passive forces that the control system utilizes (Winter et al., 1998; Federolf, 2016; Promsri et al., 2020).

## Conclusions

Several conclusions can be drawn from our results regarding the postural performance and control during bipedal standing when sensory proprioceptive and visual information were manipulated:

- Coordinative structures used to stabilize posture included not only ankle and hip in the A-P direction, but also ankle in the M-L direction, trunk rotations and knee movements specially in the more demanding conditions and for the less experienced group.
- Proprioceptive manipulation of bipedal postural control via tendon vibration engages sensory reweighting processes and postural control complexity modulations that persist even when the vibration stops.
- Differences between children and adults without gymnastics experience were found indicating that children required less use of AP ankle movements but more use of ankle ML and trunk rotations movements, larger hip and trunk rotation velocities, and larger hip accelerations but reduced ankle ML and trunk rotations accelerations. Overall, these results could be interpreted as a result of an under-developed ability of children to exploit the different sensory sources to reweight them and to stabilize posture (i.e., they have an under-developed degeneracy of the system) and higher reliance on the hip strategy.
- Gymnastics experience in the children group seemed to decrease differences produced by age (i.e., differences between children and adults without gymnastics experience), this suggests, children benefited from gymnastics experience leading to postural coordination, sensory integration, and control closer to that of adults.
- Gymnastics experience in the adult group did not seem to bring any measurable advantage when compared to adults without gymnastics experience.

## Data Availability Statement

The raw data supporting the conclusions of this article will be made available by the authors, without undue reservation.

## Ethics Statement

The studies involving human participants were reviewed and approved by Ethic Committee of Clinic Researches of the Catalan Sport Administration, Spain. Written informed consent to participate in this study was provided by the participants' legal guardian/next of kin.

## Author Contributions

AB and RA-B contributed equally to the conception of the idea and design of the study. AB, BF-U, and RA-B contributed with the implementation of the research. PF contributed by providing principal component analysis methodology and the custom-made software to implement it. All authors contributed to data analysis and writing of the manuscript.

## Conflict of Interest

The authors declare that the research was conducted in the absence of any commercial or financial relationships that could be construed as a potential conflict of interest.

## Supplementary Material

Supplementary Video 1 Animated stick figure representation of the first six principal movements (PM_1_-PM_6_) resulting from the principal component analysis (PCA).

## References

[B1] Afschrift M. Jonkers I. De Schutter J. De Groote F. (2016). Mechanical effort predicts the selection of ankle over hip strategies in nonstepping postural responses. J. Neurophysiol. 116, 1937–1945. 10.1152/jn.00127.2016 27489362 PMC5144705

[B2] Akulin V. M. Carlier F. Solnik S. Latash M. L. (2019). Sloppy, but acceptable, control of biological movement: algorithm-based stabilization of subspaces in abundant spaces. J. Hum. Kinet. 67, 49–72. 10.2478/hukin-2018-0086 31523306 PMC6714360

[B3] Alexandrov A. Frolov A. Horak F. Carlson-Kuhta P. Park S. (2005). Feedback equilibrium control during human standing. Biol. Cybern. 93, 309–322. 10.1007/s00422-005-0004-1 16228222 PMC1430400

[B4] Assaiante C. (2012). Action and representation of action during childhood and adolescence: a functional approach. Neurophysiol. Clin. Neurophysiol. 42, 43–51. 10.1016/j.neucli.2011.09.002 22200341

[B5] Assländer L. Peterka R. J. (2014). Sensory reweighting dynamics in human postural control. J. Neurophysiol. 111, 1852–1864. 10.1152/jn.00669.2013 24501263 PMC4044370

[B6] Barela J. A Jeka J. J. Clark J. E. (2003). Postural control in children. Exp. Brain Res. 150, 434–442. 10.1007/s00221-003-1441-5 12739087

[B7] Barris S. Farrow D. Davids K. (2014). Increasing functional variability in the preparatory phase of the takeoff improves elite springboard diving performance. Res. Q. Exerc. Sport 85, 97–106. 10.1080/02701367.2013.872220 24749241

[B8] Bockemühl T. Troje N. F. Dürr V. (2010). Inter-joint coupling and joint angle synergies of human catching movements. Hum. Mov. Sci. 29, 73–93. 10.1016/j.humov.2009.03.003 19945187

[B9] Busquets A. Aranda-Garcia S. Ferrer-Uris B. Marina M. Angulo-Barroso R. (2018). Age and gymnastic experience effects on sensory reweighting processes during quiet stand. Gait Posture 63, 177–183. 10.1016/j.gaitpost.2018.05.009 29763813

[B10] Busquets A. Marina M. Davids K. Angulo-Barroso R. (2016). Differing roles of functional movement variability as experience increases in gymnastics. J. Sports Sci. Med. 15, 268–276. 27274664 PMC4879440

[B11] Carver S. Kiemel T. Jeka J. J. (2006). Modeling the dynamics of sensory reweighting. Biol. Cybern. 95, 123–134. 10.1007/s00422-006-0069-5 16639582

[B12] Chen L.-C. Metcalfe J. S. Chang T.-Y. Jeka J. J. Clark J. E. (2008). The development of infant upright posture: sway less or sway differently? Exp. Brain Res. 186, 293–303. 10.1007/s00221-007-1236-1 18057920

[B13] Cuadra C. Latash M. L. (2019). Exploring the concept of iso-perceptual manifold (IPM): a study of finger force-matching tasks. Neuroscience 401, 130–141. 10.1016/j.neuroscience.2019.01.016 30673586

[B14] Cuisinier R. Olivier I. Vaugoyeau M. Nougier V. Assaiante C. (2011). Reweighting of sensory inputs to control quiet standing in children from 7 to 11 and in adults. PLoS ONE 6:e19697. 10.1371/journal.pone.0019697 21573028 PMC3090421

[B15] Daffertshofer A. Lamoth C. J. C. Meijer O. G. Beek P. J. (2004). PCA in studying coordination and variability: a tutorial. Clin. Biomech. 19, 415–428. 10.1016/j.clinbiomech.2004.01.005 15109763

[B16] de Leva P. (1996). Adjustments to Zatsiorsky-Seluyanov's segment inertia parameters. J. Biomech. 29, 1223–1230. 10.1016/0021-9290(95)00178-6 8872282

[B17] Deconinck F. De Clercq D. Van Coster R. Ooostra A. Dewitte G. Savelsbergh G. J. P.. (2007). Sensory contributions to balance in boys with developmental coordination disorder. Adapt. Phys. Act. Q. 25, 17–35. 10.1123/apaq.25.1.17 18209242

[B18] Edelman G. M. Gally J. A (2001). Degeneracy and complexity in biological systems. Proc. Natl. Acad. Sci. U.S.A. 98, 13763–13768. 10.1073/pnas.231499798 11698650 PMC61115

[B19] Eklund G. (1972). General features of vibration-induced effects on balance. Ups. J. Med. Sci. 77, 112–124. 10.1517/03009734000000016 4262735

[B20] Eklund G. Hagbarth K.-E. (1966). Normal variability of tonic vibration reflexes in man. Exp. Neurol. 16, 80–92. 10.1016/0014-4886(66)90088-4 5923486

[B21] Federolf P. Reid R. Gilgien M. Haugen P. Smith G. (2014). The application of principal component analysis to quantify technique in sports. Scand. J. Med. Sci. Sport. 24, 491–499. 10.1111/j.1600-0838.2012.01455.x 22436088

[B22] Federolf P. Roos L. Nigg B. M. (2013). Analysis of the multi-segmental postural movement strategies utilized in bipedal, tandem and one-leg stance as quantified by a principal component decomposition of marker coordinates. J. Biomech. 46, 2626–2633. 10.1016/j.jbiomech.2013.08.008 24021753

[B23] Federolf P. A. (2016). A novel approach to study human posture control: “Principal movements” obtained from a principal component analysis of kinematic marker data. J. Biomech. 49, 364–370. 10.1016/j.jbiomech.2015.12.030 26768228

[B24] Federolf P. A. Roos L. Nigg B. (2012). The effect of footwear on postural control in bipedal quiet stance. Footwear Sci. 4, 115–122. 10.1080/19424280.2012.666270

[B25] Feldman A. G. Latash M. L. (1982). Afferent and efferent components of joint position sense; interpretation of kinaesthetic illusion. Biol. Cybern. 42, 205–214. 7059622 10.1007/BF00340077

[B26] Forssberg H. Nashner L. M. (1982). Ontogenetic development of postural control in man: adaptation to altered support and visual conditions during stance. J. Neurosci. 2, 545–52. 10.1523/JNEUROSCI.02-05-00545.1982 7077364 PMC6564264

[B27] Gautier G. Thouvarecq R. Larue J. (2008a). Influence of experience on postural control: effect of expertise in gymnastics. J. Mot. Behav. 40, 400–408. 10.3200/JMBR.40.5.400-408 18782715

[B28] Gautier G. Thouvarecq R. Vuillerme N. (2008b). Postural control and perceptive configuration: influence of expertise in gymnastics. Gait Posture 28, 46–51. 10.1016/j.gaitpost.2007.09.007 17976990

[B29] Gløersen Ø. Federolf P. (2016). Predicting missing marker trajectories in human motion data using marker intercorrelations. PLoS ONE 11:e0152616. 10.1371/journal.pone.0152616 27031243 PMC4816448

[B30] Gløersen Ø. Myklebust H. Hallén J. Federolf P. (2018). Technique analysis in elite athletes using principal component analysis. J. Sports Sci. 36, 229–237. 10.1080/02640414.2017.1298826 28287028

[B31] Goble D. J. Lewis C. A Hurvitz E. A Brown S. H. (2005). Development of upper limb proprioceptive accuracy in children and adolescents. Hum. Mov. Sci. 24, 155–170. 10.1016/j.humov.2005.05.004 16043248

[B32] Goodwin G. M. Mccloskey D. I. Matthews P. B. C. (1972). The contribution of muscle afferents to keslesthesia shown by vibration induced illusionsof movement and by the effects of paralysing joint afferents. Brain 95, 705–748. 10.1093/brain/95.4.705 4265060

[B33] Hadders-Algra M. (2005). Development of postural control during the first 18 months of life. Neural Plast. 12, 99–108; discussion 263-72. 10.1155/NP.2005.99 16097478 PMC2565464

[B34] Haid T. Federolf P. (2018). Human postural control: assessment of two alternative interpretations of center of pressure sample entropy through a principal component factorization of whole-body kinematics. Entropy 20:30. 10.3390/e20010030 33265120 PMC7512231

[B35] Haid T. H. Doix A.-C. M. Nigg B. M. Federolf P. A. (2018). Age effects in postural control analyzed via a principal component analysis of kinematic data and interpreted in relation to predictions of the optimal feedback control theory. Front. Aging Neurosci. 10:22. 10.3389/fnagi.2018.00022 29459826 PMC5807376

[B36] Haid T. H. Zago M. Promsri A. Doix A.-C. M. Federolf P. A. (2019). PManalyzer: a software facilitating the study of sensorimotor control of whole-body movements. Front. Neuroinform. 13:24. 10.3389/fninf.2019.00024 31024286 PMC6461015

[B37] Hatzitaki V. Zisi V. Kollias I. Kioumourtzoglou E. (2002). Perceptual-motor contributions to static and dynamic balance control in children. J. Mot. Behav. 34, 161–170. 10.1080/00222890209601938 12057889

[B38] Hayashi R. Miyake A. Jijiwa H. Watanabe S. (1981). Postural readjustment to body sway induced by vibration in man. Exp. Brain Res. 43, 217–225. 10.1007/BF00237767 6454585

[B39] Héroux M. E. Law T. C. Y. Fitzpatrick R. C. Blouin J.-S. (2015). Cross-modal calibration of vestibular afference for human balance. PLoS ONE 10:e0124532. 10.1371/journal.pone.0124532 25894558 PMC4403994

[B40] Hirabayashi S. Iwasaki Y. (1995). Developmental perspective of sensory organization on postural control. Brain Dev. 17, 111–113. 10.1016/0387-7604(95)00009-Z 7542846

[B41] Horak F. B. Nashner L. M. Diener H. C. (1990). Postural strategies associated with somatosensory and vestibular loss. Exp. Brain Res. 82, 167–177. 10.1007/BF00230848 2257901

[B42] Hrysomallis C. (2011). Balance ability and athletic performance. Sport. Med. 41, 221–232. 10.2165/11538560-000000000-00000 21395364

[B43] Hsu W. L. Scholz J. P. Schöner G. Jeka J. J. Kiemel T. (2007). Control and estimation of posture during quiet stance depends on multijoint coordination. J. Neurophysiol. 97, 3024–3035. 10.1152/jn.01142.2006 17314243

[B44] Hwang S. Agada P. Kiemel T. Jeka J. J. (2014). Dynamic reweighting of three modalities for sensor fusion. PLoS ONE 9:e88132. 10.1371/journal.pone.0088132 24498252 PMC3909337

[B45] Jeka J. Kiemel T. Creath R. Horak F. Peterka R. (2004). Controlling human upright posture: velocity information is more accurate than position or acceleration. J. Neurophysiol. 92, 2368–2379. 10.1152/jn.00983.2003 15140910

[B46] Jeka J. Oie K. S. Kiemel T. (2000). Multisensory information for human postural control: integrating touch and vision. Exp. Brain Res. 134, 107–125. 10.1007/s002210000412 11026732

[B47] Kiemel T. Oie K. S. Jeka J. J. (2002). Multisensory fusion and the stochastic structure of postural sway. Biol. Cybern. 87, 262–277. 10.1007/s00422-002-0333-2 12386742

[B48] Kiers H. Van Dieën J. Dekkers H. Wittink H. Vanhees L. (2013). A systematic review of the relationship between physical activities in sports or daily life and postural sway in upright stance. Sport. Med. 43, 1171–1189. 10.1007/s40279-013-0082-5 23955562

[B49] Kuo A. D. Zajac F. E. (1993). Human standing posture: multi-joint movement strategies based on biomechanical constraints. Prog. Brain Res. 97, 349–358. 10.1016/S0079-6123(08)62294-3 8234760

[B50] Lamoth C. J. C. van Lummel R. C. Beek P. J. (2009). Athletic skill level is reflected in body sway: a test case for accelometry in combination with stochastic dynamics. Gait Posture 29, 546–551. 10.1016/j.gaitpost.2008.12.006 19138522

[B51] Latash M. L. (2018a). Muscle coactivation: definitions, mechanisms, and functions. J. Neurophysiol. 120, 88–104. 10.1152/jn.00084.2018 29589812 PMC6093955

[B52] Latash M. L. (2018b). Stability of kinesthetic perception in efferent-afferent spaces: the concept of iso-perceptual manifold. Neuroscience 372, 97–113. 10.1016/j.neuroscience.2017.12.018 29277305 PMC5809259

[B53] Latash M. L. (2020). Laws of nature that define biological action and perception. Phys. Life Rev. 36, 47–67. 10.1016/j.plrev.2020.07.007 32868159

[B54] Longo A. Haid T. Meulenbroek R. Federolf P. (2019). Biomechanics in posture space: properties and relevance of principal accelerations for characterizing movement control. J. Biomech. 82, 397–403. 10.1016/j.jbiomech.2018.11.031 30527635

[B55] Marin L. Bardy B. G. Bootsma R. J. (1999). Level of gymnastic skill as an intrinsic constraint on postural coordination. J. Sports Sci. 17, 615–626. 10.1080/026404199365641 10487462

[B56] Mason P. H. (2010). Degeneracy at multiple levels of complexity. Biol. Theory 5, 277–288. 10.1162/BIOT_a_00041

[B57] Mason P. H. (2015). Degeneracy: demystifying and destigmatizing a core concept in systems biology. Complexity 20, 12–21. 10.1002/cplx.21534

[B58] Mason P. H. Domínguez D. J. F Winter B. Grignolio A. (2015). Hidden in plain view: degeneracy in complex systems. Biosystems. 128, 1–8. 10.1016/j.biosystems.2014.12.003 25543071

[B59] Mattos D. J. S. Latash M. L. Park E. Kuhl J. Scholz J. P. (2011). Unpredictable elbow joint perturbation during reaching results in multijoint motor equivalence. J. Neurophysiol. 106, 1424–1436. 10.1152/jn.00163.2011 21676927 PMC3174825

[B60] McKay S. M. Wu J. Angulo-Barroso R. M. (2014). Effect of Achilles tendon vibration on posture in children. Gait Posture 40, 32–37. 10.1016/j.gaitpost.2014.02.002 24613462

[B61] Mergner T. Schweigart G. Fennell L. (2009a). Vestibular humanoid postural control. J. Physiol. Paris 103, 178–194. 10.1016/j.jphysparis.2009.08.002 19665555

[B62] Mergner T. Schweigart G. Fennell L. Maurer C. (2009b). Posture control in vestibular-loss patients. Ann. N. Y. Acad. Sci. 1164, 206–215. 10.1111/j.1749-6632.2008.03722.x 19645901

[B63] Mochizuki L. Duarte M. Amadio A. C. Zatsiorsky V. M. Latash M. L. (2006). Changes in postural sway and its fractions in conditions of postural instability. J. Appl. Biomech. 22, 51–60. 10.1123/jab.22.1.51 16760567

[B64] Nashner L. M. McCollum G. (1985). The organization of human postural movements: a formal basis and experimental synthesis. Behav. Brain Sci. 8, 135–150. 10.1017/S0140525X00020008

[B65] Ó'Reilly D. Federolf P. (2021). Identifying differences in gait adaptability across various speeds using movement synergy analysis. PLoS ONE 16:e0244582. 10.1371/journal.pone.0244582 33411749 PMC7790368

[B66] Paillard T. (2017). Plasticity of the postural function to sport and/or motor experience. Neurosci. Biobehav. Rev. 72, 129–152. 10.1016/j.neubiorev.2016.11.015 27894829

[B67] Peterka R. J. (2002). Sensorimotor integration in human postural control. J. Neurophysiol. 88, 1097–1118. 10.1152/jn.2002.88.3.1097 12205132

[B68] Peterka R. J. (2018). Sensory integration for human balance control. Handb. Clin. Neurol. 159, 27–42. 10.1016/B978-0-444-63916-5.00002-1 30482320

[B69] Peterka R. J. Black F. O. (1990). Age-related changes in human posture control: sensory organization tests. J. Vestib. Res. 1, 73–85. 1670139

[B70] Peterka R. J. Loughlin P. J. (2004). Dynamic regulation of sensorimotor integration in human postural control. J. Neurophysiol. 91, 410–423. 10.1152/jn.00516.2003 13679407

[B71] Peterson M. L. Christou E. Rosengren K. S. (2006). Children achieve adult-like sensory integration during stance at 12-years-old. Gait Posture 23, 455–463. 10.1016/j.gaitpost.2005.05.003 16002294

[B72] Pinter I. J. van Swigchem R. van Soest A. J. K. Rozendaal L. A. (2008). The dynamics of postural sway cannot be captured using a one-segment inverted pendulum model: a PCA on segment rotations during unperturbed stance. J. Neurophysiol. 100, 3197–3208. 10.1152/jn.01312.2007 18829852

[B73] Promsri A. Haid T. Federolf P. (2018). How does lower limb dominance influence postural control movements during single leg stance? Hum. Mov. Sci. 58, 165–174. 10.1016/j.humov.2018.02.003 29448161

[B74] Promsri A. Haid T. Federolf P. (2020). Complexity, composition, and control of bipedal balancing movements as the postural control system adapts to unstable support surfaces or altered feet positions. Neuroscience 430, 113–124. 10.1016/j.neuroscience.2020.01.031 32027995

[B75] Proske U. Gandevia S. C. (2012). The proprioceptive senses: their roles in signaling body shape, body position and movement, and muscle force. Physiol. Rev. 92, 1651–1697. 10.1152/physrev.00048.2011 23073629

[B76] Reimann H. Schöner G. (2017). A multi-joint model of quiet, upright stance accounts for the “uncontrolled manifold” structure of joint variance. Biol. Cybern. 111, 389–403. 10.1007/s00422-017-0733-y 28924748 PMC5688224

[B77] Reschechtko S. Cuadra C. Latash M. L. (2018). Force illusions and drifts observed during muscle vibration. J. Neurophysiol. 119, 326–336. 10.1152/jn.00563.2017 28978768 PMC5866473

[B78] Riccio G. E. (1993). Information in movement variability about the qualitative dynamics of posture and orientation. in Variability and Motor Control, eds Newell K. M. Corcos D. M. (Champaign, IL: Human Kinetics Publ), 317–357.

[B79] Ringhof S. Stein T. (2018). Biomechanical assessment of dynamic balance: specificity of different balance tests. Hum. Mov. Sci. 58, 140–147. 10.1016/j.humov.2018.02.004 29438911

[B80] Runge C. F. Shupert C. L. Horak F. B. Zajac F. E. (1999). Ankle and hip postural strategies defined by joint torques. Gait Posture 10, 161–170. 10.1016/S0966-6362(99)00032-6 10502650

[B81] Scholz J. P. Schöner G. Hsu W. L. Jeka J. J. Horak F. Martin V. (2007). Motor equivalent control of the center of mass in response to support surface perturbations. Exp. Brain Res. 180, 163–179. 10.1007/s00221-006-0848-1 17256165

[B82] Seifert L. Button C. Davids K. (2013). Key properties of expert movement systems in sport : an ecological dynamics perspective. Sports Med. 43, 167–178. 10.1007/s40279-012-0011-z 23329604

[B83] Sittig A. C. Denier van der Gon J. J. Gielen C. C. A. M. (1985). Separate control of arm position and velocity demonstrated by vibration of muscle tendon in man. Exp. Brain Res. 60, 445–453. 10.1007/BF00236930 4076369

[B84] Teasdale N. Furmanek M. P. Germain Robitaille M. de Oliveira F. C. L. Simoneau M. (2017). Sensory integration during vibration of postural muscle tendons when pointing to a memorized target. Front. Hum. Neurosci. 10:682. 10.3389/fnhum.2016.00682 28133448 PMC5233676

[B85] Thompson C. Bélanger M. Fung J. (2007). Effects of bilateral Achilles tendon vibration on postural orientation and balance during standing. Clin. Neurophysiol. 118, 2456–2467. 10.1016/j.clinph.2007.08.013 17897877

[B86] Tjernström F. Oredsson J. Magnusson M. (2006). A “wait and learn” strategy of postural control learning in children? J. Vestib. Res. 16, 257–264. 17726278

[B87] Troje N. F. (2002). Decomposing biological motion: a framework for analysis and synthesis of human gait patterns. J. Vis. 2, 371–387. 10.1167/2.5.2 12678652

[B88] van Geert P. L. C. (1994). Dynamic Systems of Development. Change Between Complexity and Chaos. Hemel Hempstead: Harvester Wheatsheaf, Simon and Schuster Ineternational Group.

[B89] Verbecque E. Vereeck L. Hallemans A. (2016). Postural sway in children: a literature review. Gait Posture 49, 402–410. 10.1016/j.gaitpost.2016.08.003 27505144

[B90] Verrel J. Lövdén M. Schellenbach M. Schaefer S. Lindenberger U. (2009). Interacting effects of cognitive load and adult age on the regularity of whole-body motion during treadmill walking. Psychol. Aging 24, 75–81. 10.1037/a0014272 19290739

[B91] Vuillerme N. Danion F. Marin L. Boyadjian A. Prieur J. M. Weise I.. (2001a). The effect of expertise in gymnastics on postural control. Neurosci. Lett. 303, 83–86. 10.1016/S0304-3940(01)01722-0 11311498

[B92] Vuillerme N. Teasdale N. Nougier V. (2001b). The effect of expertise in gymnastics on proprioceptive sensory integration in human subjects. Neurosci. Lett. 311, 73–76. 10.1016/S0304-3940(01)02147-4 11567781

[B93] Wachholz F. Kockum T. Haid T. Federolf P. (2019a). Changed temporal structure of neuromuscular control, rather than changed intersegment coordination, explains altered stabilographic regularity after a moderate perturbation of the postural control system. Entropy 21:614. 10.3390/e21060614 33267328 PMC7515107

[B94] Wachholz F. Tiribello F. Promsri A. Federolf P. (2019b). Should the minimal intervention principle be considered when investigating dual-tasking effects on postural control? Brain Sci. 10:1. 10.3390/brainsci10010001 31861521 PMC7016962

[B95] Walter C. (1998). An alternative view of dynamical systems concepts in motor control and learning. Res. Q. Exerc. Sport 69, 326–333. 10.1080/02701367.1998.10607706 9864750

[B96] Wierzbicka M. M. Gilhodes J. C. Roll J. P. (1998). Vibration-induced postural posteffects. J. Neurophysiol. 79, 143–150. 10.1152/jn.1998.79.1.143 9425185

[B97] Winter D. A. Patla A. E. Prince F. Ishac M. Gielo-Perczak K. (1998). Stiffness control of balance in quiet standing. J. Neurophysiol. 80, 1211–1221. 10.1152/jn.1998.80.3.1211 9744933

[B98] Yamagata M. Falaki A. Latash M. L. (2019). Effects of voluntary agonist-antagonist coactivation on stability of vertical posture. Motor Control 23, 304–326. 10.1123/mc.2018-0038 30612525

[B99] Yamagata M. Gruben K. Falaki A. Ochs W. L. Latash M. L. (2020). Biomechanics of vertical posture and control with referent joint configurations. J. Mot. Behav. 53, 72–82. 10.1080/00222895.2020.1723483 32041492 PMC7415538

[B100] Zago M. Pacifici I. Lovecchio N. Galli M. Federolf P. A. Sforza C. (2017). Multi-segmental movement patterns reflect juggling complexity and skill level. Hum. Mov. Sci. 54, 144–153. 10.1016/j.humov.2017.04.013 28499158

